# SGLT2 inhibitor ameliorates hypertension by regulating the CYP4A/20-HETE pathway in the kidney

**DOI:** 10.1042/BSR20260150

**Published:** 2026-09-17

**Authors:** Zhitong Zhou, Weijian Hang, Rui Zhao, Jiangang Jiang, Junfang Wu, Dao Wen Wang

**Affiliations:** 1 Division of Cardiology, Department of Internal Medicine, Tongji Hospital, Tongji Medical College, Huazhong University of Science and Technology, Wuhan, China; 2 Hubei Key Laboratory of Genetics and Molecular Mechanisms of Cardiological Disorders, Wuhan, China

**Keywords:** 20-hydroxyeicosatetraenoic acids, arachidonic acid, CYP4A, Hypertension, Sodium-Glucose Transport Protein 2 inhibitor

## Abstract

Sodium–glucose transport protein 2 inhibitors (SGLT2i), initially developed as antidiabetic agents and now established as foundational therapies for heart failure, have also shown antihypertensive effects in clinical trials involving patients with diabetes and heart failure. However, the underlying mechanisms remain incompletely understood. Given the diverse roles of arachidonic acid (AA) and its metabolites in blood pressure regulation, we investigated the antihypertensive effects of SGLT2i in hypertensive patients and an animal model and explored whether modulation of AA metabolism contributes to these effects. We first confirmed the antihypertensive effects of SGLT2i in a retrospective cohort study and spontaneously hypertensive rats (SHRs). Targeted metabolomic analysis of plasma and tissues from SHRs identified 20-hydroxyeicosatetraenoic acid (20-HETE) originating from the renal cortex as a key metabolite modulated by SGLT2i. Among the enzymes responsible for 20-HETE production, CYP4A but not CYP4F was found to be down-regulated by dapagliflozin at both mRNA and protein levels. Immunofluorescence colocalization further localized this effect to proximal tubular epithelial cells, where SGLT2i reduced CYP4A expression and subsequent 20-HETE production, leading to attenuated renal inflammation, fibrosis, and blood pressure elevation. Together, these findings not only confirm the antihypertensive effects of SGLT2i but also delineate a novel antihypertensive mechanism by which lower blood pressure, demonstrating that modulation of AA metabolism contributes partially to their blood pressure-lowering effects.

## Introduction

Hypertension remains one of the greatest global public health challenges because of its increasing prevalence and persistently low rates of blood pressure control [1,2]. A recent report revealed that the global population of adults aged 30–79 years with hypertension doubled between 1990 to 2019 [3], yet only 21% achieved effective blood pressure control [4]. Despite the availability of comprehensive antihypertensive therapies [5], the persistently low control rates and the substantial global burden underscore the urgent need for further investigation into the pathogenesis of hypertension and the development of novel therapeutic strategies.

Sodium–glucose cotransporter 2 inhibitors (SGLT2i), originally developed as antidiabetic agents, have attracted considerable attention for their protective effects beyond glycemic control. Landmark cardiovascular outcome trials, including DECLARE–TIMI 58 [6], EMPA-REG OUTCOME [7,8], and CANVAS [9], all reported cardiovascular and renal benefits of SGLT2i in patients with type 2 diabetes. These findings prompted further investigation in patients with heart failure and chronic kidney disease. The DAPA-HF [10] and EMPEROR-Reduced [11] trials demonstrated the efficacy of SGLT2i in heart failure with reduced ejection fraction [12], while subsequent studies extended these benefits to heart failure with preserved ejection fraction [13]. In parallel, the DAPA-CKD [14] and EMPA-KIDNEY [15] studies highlighted the therapeutic potential of SGLT2i in chronic kidney disease.

Across these clinical trials, SGLT2i were consistently observed to exert beneficial effects on blood pressure reduction. For example, in the DECLARE–TIMI 58 trial [6], diabetic patients treated with dapagliflozin exhibited lower blood pressure compared with those receiving placebo. Similarly, the EMPA-REG OUTCOME trial [16] demonstrated that empagliflozin reduced blood pressure in diabetic patients independently of baseline estimated glomerular filtration rate. Moreover, in the DAPA-HF trial [10], the dapagliflozin group showed a significant reduction in SBP compared with the placebo in patients with heart failure. Nevertheless, clinical trials specifically designed to evaluate the antihypertensive effects of SGLT2i in hypertensive populations remain limited. Furthermore, a deeper understanding of the underlying mechanisms by which SGLT2i lower blood pressure is essential to support their broader clinical application in hypertension management.

Arachidonic acid (AA), an ω-6 polyunsaturated fatty acid, is a key component of cell membrane phospholipids [17]. Its metabolites have been implicated in cardiovascular biology, carcinogenesis, and various inflammatory diseases, including asthma and arthritis [18]. Importantly, these metabolites also play critical roles in hypertension. Prostaglandins, derived from AA via cyclooxygenase pathways, have been reported to influence vascular tone by inducing either vasodilation or vasoconstriction, thereby regulating blood pressure [19]. Epoxyeicosatrienoic acids, produced from AA by cytochrome P450 (CYP) enzymes, contribute to the preservation of endothelial function and blood pressure reduction [20]. In contrast, 20-hydroxyeicosatetraenoic acid (20-HETE), another CYP-derived AA metabolite, exerts context-dependent effects on blood pressure: it can promote hypertension through binding to G-protein receptor 75 [21], while also exhibiting antihypertensive potential via modulation of sodium excretion [22]. Given the diverse roles of AA metabolites in blood pressure regulation, it is crucial to investigate whether they contribute to the antihypertensive effects of SGLT2i and whether they may serve as potential therapeutic targets.

In the present study, we retrospectively evaluated the blood pressure effects of SGLT2i in hypertensive patients at Tongji Hospital by comparing blood pressure levels between those treated with and without SGLT2i. We further investigated the antihypertensive effects of SGLT2i in a spontaneously hypertension rat (SHR) model, examined their impact on AA metabolism, and elucidated the underlying mechanism involved in blood pressure reduction.

## Materials and methods

### Study population and design

**Figure 1:**
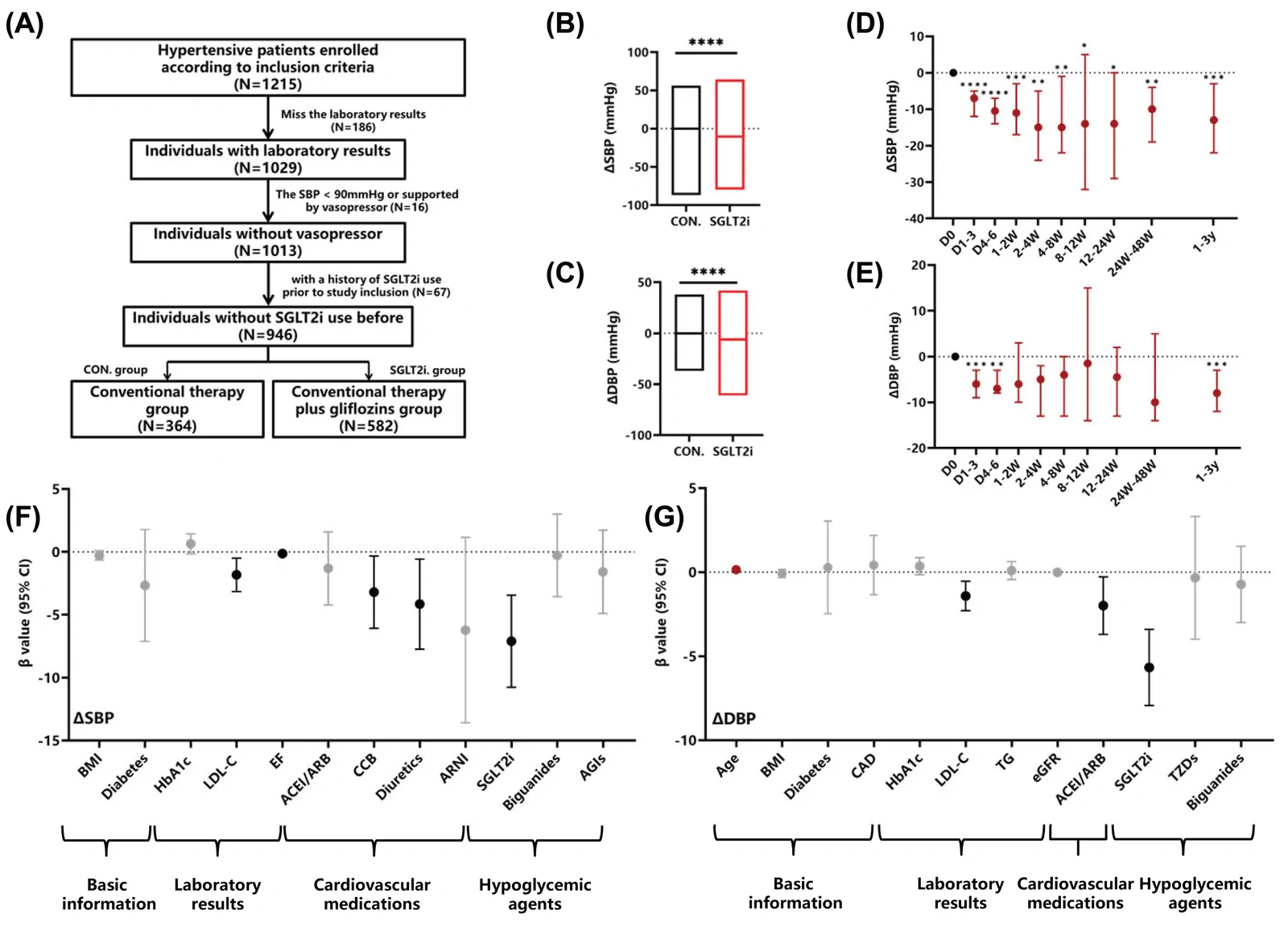
SGLT2i decrease SBP and DBP in patients with hypertension (**A**) Flowchart of the screening process for the retrospective cohort study. (**B**) ΔSBP (final SBP – initial SBP) and (**C**) ΔDBP (final DBP – initial DBP) in the conventional therapy group and conventional therapy plus gliflozins group. (**D**) ΔSBP and (**E**) ΔDBP in the conventional therapy group (day 0) and the conventional therapy plus gliflozins group stratified by duration of SGLT2 inhibitor use. (**F,G**) Generalized linear models of ΔSBP and ΔDBP. *P<0.05, **P<0.01, ***P<0.001, ****P<0.0001.

This retrospective cohort study assessed the antihypertensive effects of SGLT2 inhibitors in hypertensive patients at Tongji Hospital (2013–2023). Using electronic medical records from the YiduCloud platform, we identified 946 eligible patients who were divided into two groups: conventional therapy alone (CON, *n* = 364) and conventional therapy plus gliflozins (SGLT2i, *n* = 582) (Figure 1A). The inclusion criteria of the retrospective cohort were as follows: (1) age ≥18 years; (2) patients with a history of hypertension or newly diagnosed with hypertension, based on the 2020 Global Hypertension Practice Guidelines approved [23]; (3) complete medical records of antihypertensive medication use; and (4) no changes to antihypertensive therapy during the blood pressure monitoring period. Patients were excluded if they met any of the following criteria: (1) incomplete blood pressure monitoring data (at least two valid recordings required); (2) incomplete laboratory indices; (3) systolic blood pressure (SBP) below 90 mmHg or requirement for vasopressor support; or (4) prior use of SGLT2i before study enrollment.

Blood pressure analysis was based on initial and final recorded values. In the conventional therapy plus gliflozins group, initial blood pressure was defined as the last recorded measurement before initiation of gliflozin treatment, and the final blood pressure as the last recorded measurement before discontinuation. In the conventional therapy group, the initial and final blood pressure measurements were defined as the first and last recorded measurements during the study period, during which no adjustments were made to antihypertensive medications.

The study was approved by the Research Ethics Committee of Tongji Medical College (TJ-IRB20220604), with a waiver of informed consent due to its retrospective and anonymous nature of the analysis.

### Animal experiments

Six-week-old male SHR and Wistar-Kyoto (WKY) rats were purchased from Beijing Charles River Laboratories. All rats were bred at the Laboratory Animal Center of Tongji Medical College under specific pathogen-free conditions. They were housed in temperature-controlled cages with a 12-h light/dark cycle and had free access to water and a standard chow diet. After a two-week acclimation period, SHRs were randomly assigned to either the hypertension group (SHR group, *n* = 8) or the hypertension plus dapagliflozin group (SHR-D group, *n* = 6), WKY rats served as the normotensive control group (WKY group, *n* = 4–5, one rat died during echocardiographic examination due to anesthesia). In the SHR-D group, dapagliflozin (1 mg/kg) [24,25] (diluted in 0.2% DMSO and 99.8% ddH_2_O) was administered daily via oral gavage. This dosage is comparable to the standard clinical dose of 10 mg daily in human [26]. Rats in the WKY and SHR groups received the equivalent volume of vehicle. Blood pressure was measured biweekly throughout the study. After 34 weeks of intervention—the time point at which blood pressure trends stabilized over three consecutive measurements—rats were deeply anesthetized with 2% pentobarbital sodium (50 mg/kg, i.p.). They were then killed by exsanguination via the abdominal aorta for plasma collection, after which tissues were harvested. All experimental procedures were approved by the Institutional Animal Research Committee of Tongji Medical College (No. 2021-3761).

### Blood pressure and fasting glucose measurement

Blood pressure was measured using the tail-cuff method with a noninvasive blood pressure system (Softron Biotechnology, Beijing, China). To ensure accuracy, animals were placed in a suitable restrainer with their tail naturally extended and warmed for 10 min before each measurement. Once the rats were calm, six measurement cycles were performed, and the final value was calculated as the mean of these readings. All measurements were conducted at the same time of day under consistent environmental conditions.

Fasting blood glucose was measured at 12 and 42 weeks from tail tip blood samples using a CONTOUR®TS meter (Ascensia Diabetes Care, Shanghai, China) to assess the short- and long-term effects of SGLT2i on glucose levels. Following an overnight fast, the tail tip was sterilized with alcohol, and a sterile lancet was used to gently puncture the tail tip to obtain a small volume of capillary blood. The first drop of blood was discarded, and the second drop was applied to the test strip, after which the glucometer automatically displayed the glucose value. After blood collection, the puncture site was immediately pressed for at least 30 s until hemostasis was achieved, and the rat was returned to its cage only after confirming that active bleeding had ceased.

### Echocardiography

Echocardiograph was performed one day before sacrifice at 42 weeks using a 30-MHz high-frequency scan head (Vevo770; VisualSonics, Toronto, ON, Canada). Briefly, rats were anesthetized with isoflurane (induction: 3%–5%; maintenance: 1.5%–2%) in 100% oxygen, positioned supine on a platform, and secured to the electrode plate. M-mode images of the short- and long-axis views of the left ventricle were recorded for subsequent analysis of wall thickness, ejection fraction, and chamber dimensions using the Vevo770 integrated software (VisualSonics, Toronto, ON, Canada).

### Cell culture and treatment

HK-2 cells were cultured in DMEM/F12 medium supplemented with 10% fetal bovine serum. Upon reaching appropriate confluence, cells were treated for 24 h with various agents: blank control, Angiotensin II (AngII; 1 μM; MedChemExpress, U.S.A.), AngII (1 μM) combined with dapagliflozin (2.5 μM; BioVision, U.S.A.), or dapagliflozin (2.5 μM) alone. For knockdown experiments, HK-2 cells were transfected with si-CYP4A11 or si-NC (all synthesized by GenePharma, Shanghai, China) using the CALNP™ RNAi *in vitro* transfection reagent (D-Nano Therapeutics, Beijing, China) for 24 h. After transfection, the culture medium was replaced with fresh medium, and cells were subsequently treated with AngII (1 μM), dapagliflozin (2.5 μM), or 20-HETE (1 μM; TargetMol Chemicals Inc., Boston, MA, U.S.A.) for 24 h.

### Targeted metabolic analysis for AA metabolites

Metabolic profiling of AA metabolites from the COX, LOX, and CYP pathways was performed using liquid chromatography–tandem mass spectrometry (LC-MS/MS) following a previously optimized method [27]. Briefly, 100 μl of plasma was extracted via solid-phase extraction using conditioned Waters Oasis-HLB cartridges. Samples and internal standard mixtures were loaded onto the cartridges, and the elute was dried under nitrogen flow before reconstitution in 100 μl of methanol. For tissue samples, 6–8 mg of renal cortex, heart, or aorta tissue was extracted with 600 μl of precooled methanol containing 10 μl of internal standard mixture. Tissues were homogenized using a tissue lyser for three cycles of 90 s at 4°C. The supernatant was dried under nitrogen flow and dissolved in 1 ml of 5% methanol, loaded onto the cartridge, and processed following the same protocol as plasma samples. LC-MS/MS acquisition was carried out on an AB SCIEX Triple Quad 6500+ mass spectrometer (AB SCIEX, U.S.A.) equipped with an electrospray ionization source operating in negative ion mode. Chromatographic separation was achieved on a UPLC BEH C18 column (1.7 μm, 50 × 2.1 mm i.d.) maintained at 40°C, with an injection volume of 10 μl. Mass spectrometry was performed using a multiple reaction monitoring system, with MilliQ water containing 0.01% CH_3_COOH (A) and acetonitrile containing 0.01% CH_3_COOH (B) as the mobile phase. Each metabolite was identified by its parent ion and daughter ion selected from each standard during method development, aided by retention times. Quantification was performed against the area of the internal standards using an OS station, and results were expressed as pg/ml of plasma or per milligram of tissue.

### RNA extraction and real-time PCR

Total RNA was extracted from frozen tissues or cells using TRIzol reagent (TaKaRa, Shiga, Japan) and dissolved in DEPC-treated water. RNA concentrations were measured using a microspectrophotometer (Kaiao Technology, Beijing, China). Reverse transcription was performed with 1 μg of RNA using the HiScript II Q RT SuperMix for qPCR (Vazyme, Nanjing, China) according to the manufacturer’s instructions.

Real-time PCR was carried out in a 10 μl reaction system containing 5 nM each of forward and reverse primers (Supplementary Table S1), 5 μl Hieff® qPCR SYBR Green Master Mix (Yeason, Shanghai, China), 1.0 μl or 2.0 μl of cDNA, and DEPC-treated water to adjust the final volume. Amplification was performed on a 7900HT Fast Real-Time PCR system (Applied Biosystems, Foster City, CA). Relative mRNA expression levels were calculated using the 2−ΔΔCt method and normalized to GAPDH, with the average expression in the control group set as the reference.

### Western blotting

Western blot analysis was performed using renal cortical tissuelysates or celllysates. After protein separation by SDS–PAGE and transfer onto PVDF membranes, the membranes were blocked with 5% BSA in TBST for 1 h and incubated overnight at 4°C with primary antibodies against CYP4A (E-6; Santa Cruz Biotechnology, U.S.A.), CYP4A11 (Proteintech, China), NHE3 (SLC9A3/NHE3 antibody, Proteintech, China), COL1A1 (ABclonal Biotechnology Co., China), and GAPDH (Proteintech, China). Membranes were then washed six times with TBST and incubated with an HRP-conjugated secondary antibody for 1 h at room temperature. After another six TBST washes, protein bands were visualized using chemiluminescence (BOSTER Biological Technology, Wuhan, China) and the ECL system (Beyotime Institute of Biotechnology, Nanjing, China). Protein expression levels were quantified relative to GAPDH as the loading control, with the control group used as the reference.

### Measurement of IL-1β, IL-6, and TNF-α levels

Levels of IL-1β, IL-6, and TNF-α in tissue homogenates and cell culture supernatants were measured using enzyme-linked immunosorbent assay (ELISA) kits (Bioswamp, Wuhan, China) according to the manufacturer’s instructions. Levels of COL1A1 in cell culture supernatants were also measured using ELISA kits (ELK Biotechnology, Wuhan, China) according to the manufacturer’s instructions.

### Histology examination

Rat hearts and kidneys were dissected, fixed in 4% paraformaldehyde, and embedded in paraffin. Tissues were sectioned at 4 μm thickness. Wheat germ agglutinin (WGA) staining and Masson’s trichrome staining were performed to assess hypertrophy and fibrosis, respectively.

### Immunofluorescence

Paraffin sections of rat kidney were baked at 60°C for 1 h, deparaffinized in xylene, and rehydrated through a graded ethanol series. Antigen retrieval was performed using Tris-EDTA Antigen Retrieval Solution (Servicebio, Wuhan, China). After cooling and PBS washing, sections were treated with an autofluorescence quenching agent (Servicebio, Wuhan, China). Blocking was performed with 5% BSA in 0.3% Triton X-100, followed by overnight incubation at 4°C with primary antibodies (CYP4A Antibody (E-6), Santa Cruz Biotechnology, U.S.A.; SGLT2 Antibody, Proteintech, China; SLC9A3/NHE3 antibody, Proteintech, China). After PBS washing, sections were incubated for 2 h in the dark with a secondary antibody mixture conjugated with Dylight 594 and AlexaFluor value (kcal/mol) representing inte488 (Abbkine, Wuhan, China). Nuclei were stained with DAPI (Servicebio, Wuhan, China). Following another treatment with the tissue autofluorescence quencher, sections were washed, mounted with antifade mounting medium, and imaged under a fluorescence microscope.

### Molecular docking

The SGLT2i compound name, molecular weight, and 3D structure of the gliflozins were obtained from the PubChem database (https://pubchem.ncbi.nlm.nih.gov/). The 3D structure of CYP4A was retrieved from the RCSB PDB database (http://www.rcsb.org/). Using AutoDock software, ligands and proteins were prepared for molecular docking by removing water molecules, adding hydrogen atoms, and making necessary amino acid modifications. Energy minimization was performed, and the force field parameters were optimized to achieve low-energy conformations. Molecular docking was then conducted between CYP4A and each gliflozin, with the binding affinity value (kcal/mol) representing interaction strength—lower values indicating more stable interaction. Docking results were further analyzed and visualized using Discovery Studio and PyMOL software.

### Statistical analysis

Statistical analysis was performed using SPSS Statistics 25.0 and GraphPad Prism 9.3.0. Data distribution and homoscedasticity were assessed using the Shapiro–Wilk or D'Agostino–Pearson test. For the retrospective cohort study, continuous variables were expressed as the median and interquartile range (IQR). The Mann–Whitney *U* test was used to compare changes in systolic and diastolic blood pressure between the two groups, while Dunn’s test was applied for multiple group comparisons. Categorical variables were expressed as counts and percentages and analyzed using the chi-square test or Yates’s continuity correction. Generalized linear models were used to assess the relationship between independent and dependent variables, with results reported as β values with 95% confidence intervals. Propensity score matching (PSM) in a 1:1 ratio was used to achieve balanced covariate distribution between the control and gliflozin groups. Balance was assessed using the absolute standardized mean difference (SMD), with values of SMD <0.2 considered to indicate acceptable balance.

For the animal experiments, normally distributed continuous variables were expressed as mean ± SD and compared using Tukey’s HSD test; non-normally distributed variables were expressed as median (IQR) and analyzed using Dunn’s test. For cellular experiments, prespecified pairwise comparisons were performed using Šídák’s test for normally distributed data or Dunn’s test for non-normally distributed data. A two-tailed *P* -value <0.05 was considered statistically significant.

## Results

### Demographic data and clinical characteristics

The retrospective cohort study included 946 patients with hypertension, whose baseline characteristics are summarized in Supplementary Table S2. No significant differences in age or sex distribution were observed between the two groups. However, BMI and the prevalence of diabetes were significantly higher in the conventional therapy plus gliflozins group than in the conventional therapy group (*P*<0.001). Conversely, the prevalence of cardiomyopathy was higher in the conventional therapy group (*P* = 0.041). No significant differences were observed in other cardiovascular comorbidities.

Treatment regimens also differed between the two groups (Supplementary Table S2). With the exception of dipeptidyl peptidase-4 inhibitors and glinides, hypoglycemic agents were more frequently prescribed in the conventional therapy plus gliflozins group. In contrast, cardiovascular medications such as alpha-adrenergic blockers, beta-adrenergic blockers, and diuretics were more commonly used in the conventional therapy group, whereas the use of angiotensin receptor-neprilysin inhibitors was relatively low in this group.

### SGLT2i reduce blood pressure in hypertensive patients

To assess the antihypertensive effect of SGLT2i, we compared changes in systolic and diastolic blood pressure between the two groups. As shown in Figure 1B, the reduction in SBP (ΔSBP) was significantly greater in the SGLT2i group (IQR: −11 (−25–2) mmHg) relative to the CON group (IQR: 0 (−10–2) mmHg). Similarly, the reduction in DBP (ΔDBP) was more obvious in the SGLT2i group than in the CON group (Figure 1C). Given the distribution of specific SGLT2i used (dapagliflozin: 112/582; empagliflozin: 264/582; canagliflozin: 206/582), we further evaluated the antihypertensive effects of each agent. All gliflozin-treated subgroups demonstrated statistically significant reductions in both SBP and DBP compared with the CON group (Supplementary Figure S1). Moreover, the SBP reduction induced by SGLT2i was sustained over time (Figure 1D), and a significant DBP reduction was observed in both the short- and long-term treatment periods (Figure 1E).

To adjust for covariate differences and better assess the antihypertensive effect of SGLT2i, we applied a multivariate generalized linear model to examine the relationship between blood pressure changes and potential confounding, including gliflozins use and baseline variables identified by univariate analysis. SGLT2i treatment exerted a significant effect on ΔBP (ΔSBP: *P<*0.001; ΔDBP: *P<*0.001), with covariate-adjusted estimated reductions of 7.1 mmHg for SBP and 5.7 mmHg for DBP (Figure 1F,G and Supplementary Tables S3 and S4). Furthermore, PSM was performed on the original cohort, yielding 145 matched pairs for in-depth analysis. After matching for multiple parameters, significant differences in ΔSBP and ΔDBP between the two groups persisted (Table 1). Collectively, these results indicate that gliflozins provide additional blood pressure reduction beyond conventional therapy.

**Table 1:** Baseline characteristics of the conventional therapy and conventional therapy plus gliflozins groups after PSM

	Conventional therapy (*n* = 145)	Conventional plus gliflozins (*n* = 145)	SMD	*P*-value
**Basic information**				
Age, years	62 (52–71)	63 (56–67)	0.040	0.554
Sex, male (%)	89 (61.4)	88 (60.7)	−0.014	0.904
BMI (kg/m^2^)	24.2 (22.5–26.1)	24.6 (22.6–27.0)	0.120	0.196
Smoking (%)	39 (26.9)	31 (21.4)	−0.129	0.272
Drinking (%)	21 (14.5)	15 (10.3)	−0.128	0.285
Diabetes (%)	138 (95.2)	138 (95.2)	0.000	>0.999
CAD (%)	60 (41.4)	52 (35.9)	−0.113	0.335
Cardiomyopathy (%)	6 (4.1)	4 (2.8)	−0.071	0.520
Arrhythmia (%)	16 (11.0)	15 (10.3)	−0.023	0.849
**Laboratory results**				
HbA1c (%)	6.80 (6.20–8.45)	7.40 (6.65–8.60)	0.093	**0.006**
LDL-C (mmol/l)	2.29 (1.64–3.03)	2.19 (1.56–2.97)	0.025	0.708
HDL-C (mmol/l)	0.96 (0.83–1.17)	0.99 (0.84–1.19)	0.045	0.603
TG (mmol/l)	1.55 (1.11–2.61)	1.58 (1.11–2.28)	−0.004	0.963
eGFR (ml/min/1.73 m^2^)	84.0 (59.7–98.4)	79.1 (61.7–97.5)	0.055	0.625
EF (%)	64.0 (59.5–68.0)	65.0 (60.0–68.0)	0.098	0.504
**Treatment**				
**Cardiovascular medications (*n*, %)**				
Alpha-blockers	1 (0.7)	0 (0.0)	−0.119	>0.999
ACEI/ARB	51 (35.2)	55 (37.9)	0.056	0.626
Beta-blockers	52 (35.9)	48 (33.1)	−0.059	0.621
CCB	58 (40.0)	60 (41.4)	0.029	0.811
Diuretics	33 (22.8)	26 (17.9)	−0.122	0.307
ARNI	4 (2.8)	3 (2.1)	−0.045	>0.999
Anticoagulant drugs	15 (10.3)	18 (12.4)	0.066	0.579
Antiplatelet drugs	75 (51.7)	77 (53.1)	0.028	0.814
Lipid-lowering agents	96 (66.2)	95 (65.5)	−0.015	0.901
**Hypoglycemic agents (*n*, %)**				
SUs	7 (4.8)	9 (6.2)	0.061	0.607
TZDs	5 (3.4)	8 (5.5)	0.102	0.395
Biguanides	33 (22.8)	35 (24.1)	0.031	0.782
DPP-4 inhibitors	24 (16.6)	28 (19.3)	0.070	0.540
AGIs	58 (40.0)	62 (42.8)	0.057	0.633
Glinides	3 (2.1)	4 (2.8)	0.045	>0.999
GLP-1 agonists	7 (4.8)	6 (4.1)	−0.034	0.777
Insulin	65 (44.8)	64 (44.1)	−0.014	0.906
**Baseline blood pressure**				
**Initial blood pressure**				
SBP (mmHg)	134 (120–146)	137 (123–154)	-	0.072
DBP (mmHg)	77 (70–84)	82 (75–91)	-	**<0.001**
**Final blood pressure**				
SBP (mmHg)	129 (120–142)	126 (119–137)	-	0.107
DBP (mmHg)	76 (70–84)	76 (70–83)	-	0.864
**ΔBP**				
ΔSBP	0 (−14–2)	−10 (−27–1)	-	**<0.001**
ΔDBP	0 (−6–6)	−6 (−14–2)	-	**<0.001**

Data were presented as *n* (%) or median with interquartile range (Q1–Q3).

Ābbreviations used: BMI, body mass index; CAD, coronary heart disease; HbA1c, glycated hemoglobin/hemoglobin A1c; LDL-C, low-density lipoprotein cholesterol; HDL-C, high density lipoprotein cholesterol; TG, triglyceride; eGFR, estimated glomerular filtration rate; EF, ejection fraction; Alpha blockers, alpha-adrenergic blockers; ACEI, angiotensin converting enzyme inhibitor; ARB, angiotensin receptor blocker; Beta-blockers, beta-adrenergic blockers; CCB, Calcium channel blockers; ARNI, angiotensin receptor-neprilysin inhibitors; SUs, sulfonylureas; TZDs, thiazolidinediones; DPP-4, dipeptidyl peptidase IV; AGIs, Alpha-glucosidase inhibitors; GLP-1, glucagon-like peptide-1; SBP, systolic blood pressure; DBP, diastolic blood pressure. ΔSBP (Final SBP – Initial SBP); ΔDBP (Final DBP – Initial DBP).

### Dapagliflozin reduced SBP in spontaneously hypertensive rats

**Figure 2:**
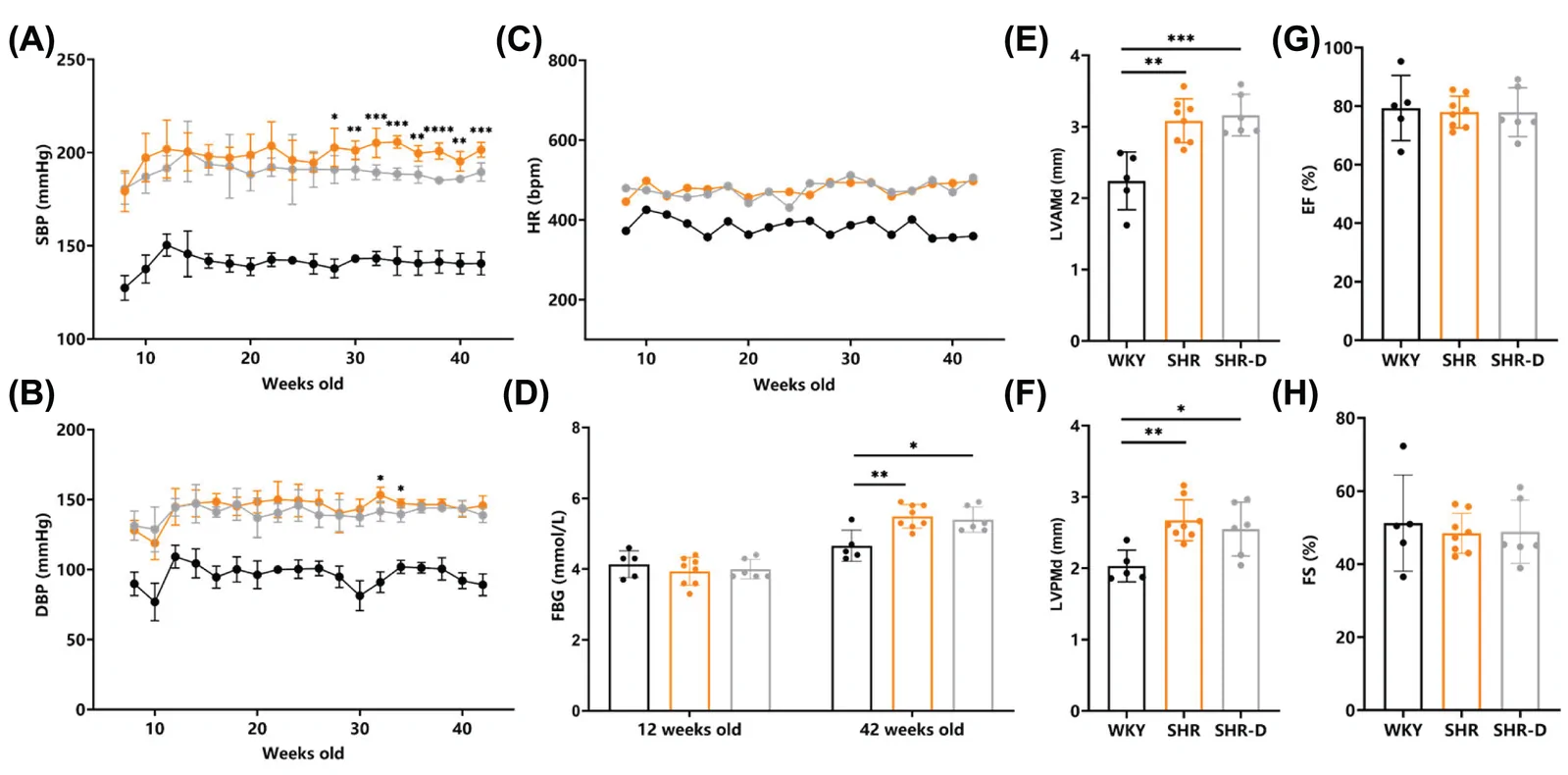
Dapagliflozin lowers SBP in spontaneously hypertensive rats (**A**) SBP, (**B**) DBP, (**C**) HR of rats in different groups. (**D**) The fasting blood glucose of rats at 12 and 42 weeks old. (**E-H**) diastolic left ventricular anterior wall (LVAMd), diastolic left ventricular posterior wall (LVPMd), Ejection fraction, and fractional shortening of rats at 42 weeks old. **Keys**: Control group (WKY), hypertension group (SHR), hypertension plus dapagliflozin group (SHR-D). *P<0.05, **P<0.01, ***P<0.001, ****P<0.0001. Multiple comparisons were performed using Tukey’s HSD test for normally distributed data or Dunn’s test for non-normally distributed data.

To further investigate the consistent antihypertensive effect of gliflozins observed in our retrospective cohort study—an effect independent of confounding variables—we established an SHR model, with WKY rats serving as normotensive controls. Prior to dapagliflozin intervention, the SBP, DBP, and heart rate (HR) were compared between the SHR and SHR-D (SHR + dapagliflozin) groups, both of which exhibited significantly higher values than the WKY control group (Figure 2A–C).

Following dapagliflozin intervention initiated at 8 weeks of age, a statistically significant reduction in SBP was observed in the SHR-D group beginning at 28 weeks of age, and this effect was sustained throughout subsequent biweekly measurements (Figure 2A). Fasting glucose levels were measured at 12 and 42 weeks of age to evaluate both short- and long-term glycemic effects of dapagliflozin (Figure 2D). Short-term treatment did not affect fasting glucose in SHRs. At the long-term assessment, although fasting glucose was higher in both SHR and SHR-D groups than in the WKY group, no significant difference was observed between the two hypertensive groups.

At the end of the experiment, echocardiography showed no significant differences in ejection fraction or fractional shortening among the three groups (Figure 2G,H). Both left ventricular anterior wall and posterior wall thicknesses in diastole were significantly greater in the SHR and SHR-D groups than in the WKY group, with no significant difference between the two hypertensive groups (Figure 2E,F). Consistent with these findings, WGA staining showed that the mean cross-sectional area of cardiomyocytes was significantly larger in both hypertensive groups than in the WKY group, with no significant difference between the SHR and SHR-D groups (Supplementary Figure S2A,B). In contrast, Masson's trichrome staining of heart sections indicated that the fibrosis area fraction was significantly higher in the SHR group than in the WKY group, and this increase was significantly attenuated by dapagliflozin treatment in the SHR-D group (Supplementary Figure S2A,C).

### Dapagliflozin decreases plasma 20-HETE levels that originated from renal cortex

**Figure 3:**
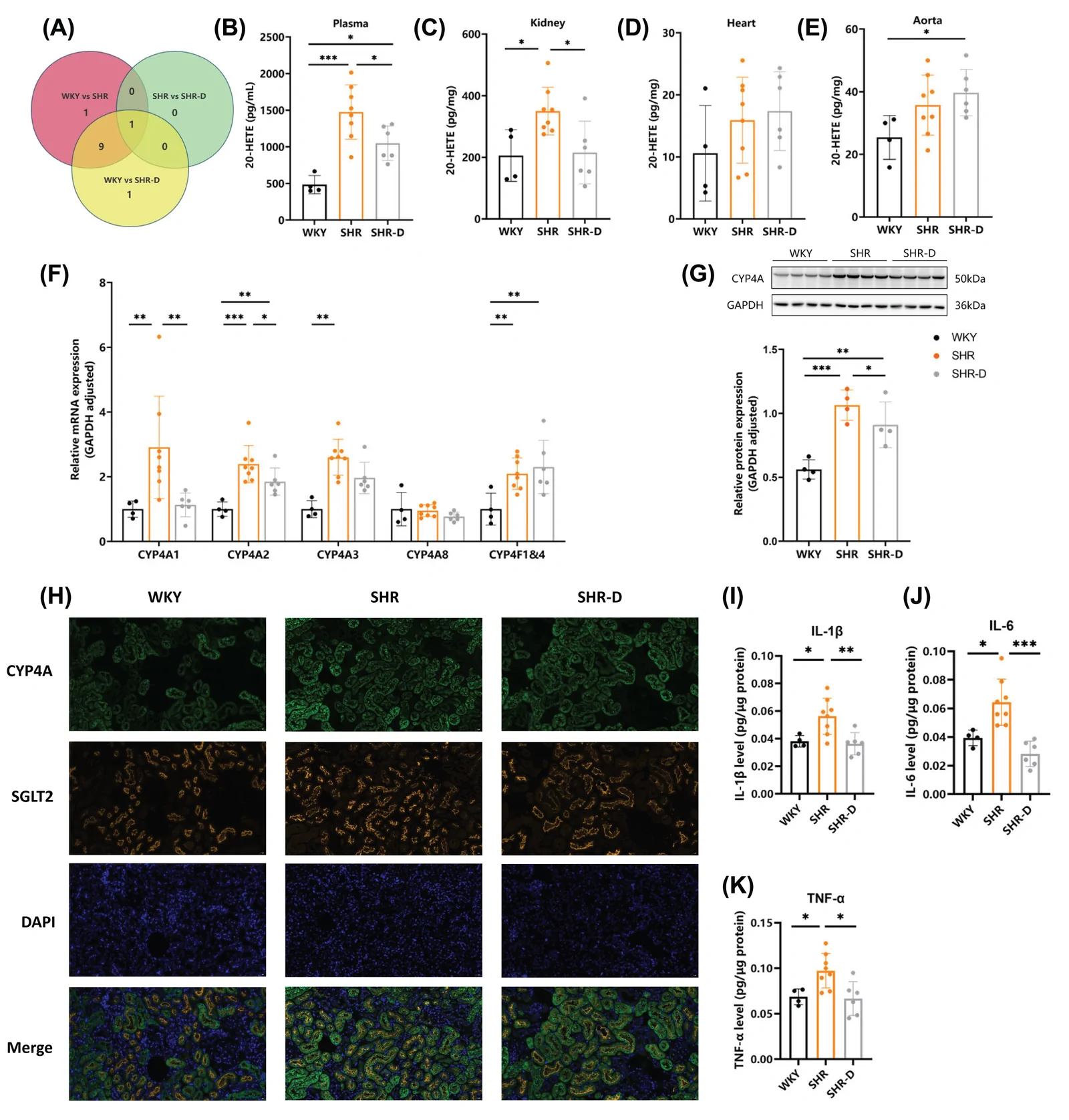
Dapagliflozin suppresses CYP4A expression and 20-HETE levels in the renal cortex of SHRs (**A**) The Venn plot of concentrations of AA metabolites in plasma of rats. The 20-HETE levels of rats in (**B**) plasma, (**C**) kidney (renal cortex), (**D**) heart, and (**E**) aorta. (**F**) Relative mRNA expression levels of CYP4A and CYP4F in the renal cortex of rats. (**G**) Relative protein expression levels of CYP4A in renal cortex of rats. (**H**) Representative images of immunofluorescence staining of the renal cortex in WKY, SHR, and SHR-D groups. (**I–K**) Protein levels of IL-1β, IL-6, and TNF-α in the renal cortex of WKY, SHR, and SHR-D groups, determined by ELISA and normalized to total protein concentration. Control group (WKY), hypertension group (SHR), hypertension plus dapagliflozin group (SHR-D). Multiple comparisons were performed using Tukey’s HSD test for normally distributed data or Dunn’’s test for non-normally distributed data. *P<0.05, **P<0.01, ***P<0.001, ****P<0.0001.

To explore the mechanism underlying the antihypertensive effect of dapagliflozin, we performed targeted metabolomic analysis of AA metabolites in rat plasma. Compared with the WKY group, both the SHR and SHR-D groups exhibited significantly elevated levels of several metabolites, including dihydroxy-eicosatetraenoic acids (DHETs: 5,6-DHET, 8,9-DHET, 11,12-DHET, and 14,15-DHET), hydroxyeicosatetraenes (HETEs: 12-HETE and 20-HETE), and polyunsaturated fatty acids (AA, DHA, and LA) (Supplementary Table S5). Among these, only plasma 20-HETE levels were significantly reduced in the SHR-D group compared with the SHR group (Figure 3A,B).

To identify the tissue source of this reduction, we measured 20-HETE levels in organs commonly involved in hypertension pathophysiology—kidney, heart, and aorta (Figure 3C–E). Given that the AA omega-hydroxylation occurs primarily in the renal cortex [28], we specifically analyzed cortical tissue. While 20-HETE concentrations in the heart and aorta were relatively low and did not differ significantly among groups, renal cortical 20-HETE levels were markedly lower in dapagliflozin-treated SHRs (215.5 ± 101.8 pg/ml) than in untreated SHRs (350.0 ± 77.5 pg/ml), suggesting the renal cortex as the target organ for SGLT2i-mediated modulation of 20-HETE metabolism.

Because 20-HETE is primarily synthesized by CYP4A and CYP4F ω-hydroxylases [29], we assessed the expression levels of these enzymes in the renal cortex. RT-PCR revealed that mRNA levels of CYP4A1 and CYP4A2 were significantly higher in the SHR group than in both the WKY and SHR-D groups (Figure 3F). Consistently, Western blot analysis showed elevated CYP4A protein expression in the SHR group, which was reduced by dapagliflozin treatment (Figure 3G). These findings indicate that dapagliflozin suppresses CYP4A transcription and translation in the renal cortex.

Immunofluorescence co-staining confirmed the colocalization of CYP4A and SGLT2, which is predominantly expressed in proximal tubular cells (Figure 3H). Furthermore, dapagliflozin treatment attenuated renal inflammation, as evidenced by reduced levels of IL-1β, IL-6, and TNF-α in the rat renal cortex (Figure 3I–K). Masson staining also revealed that dapagliflozin reduced fibrosis in the renal cortex of SHRs (Supplementary Figure S3D,E). Based on these findings, we hypothesize that dapagliflozin specifically inhibits CYP4A expression in proximal tubular epithelial cells. Taken together, we conclude that SGLT2i lower blood pressure in SHRs by inhibiting the CYP4A/20-HETE pathway and that this effect primarily involves the chronic actions of 20-HETE on hypertension—namely, renal inflammation and fibrosis—which may explain the delayed antihypertensive effect of dapagliflozin observed in our animal study.

### Dapagliflozin inhibits AngII-induced inflammation and CYP4A11/20-HETE secretion in proximal renal tubular epithelial cells

**Figure 4:**
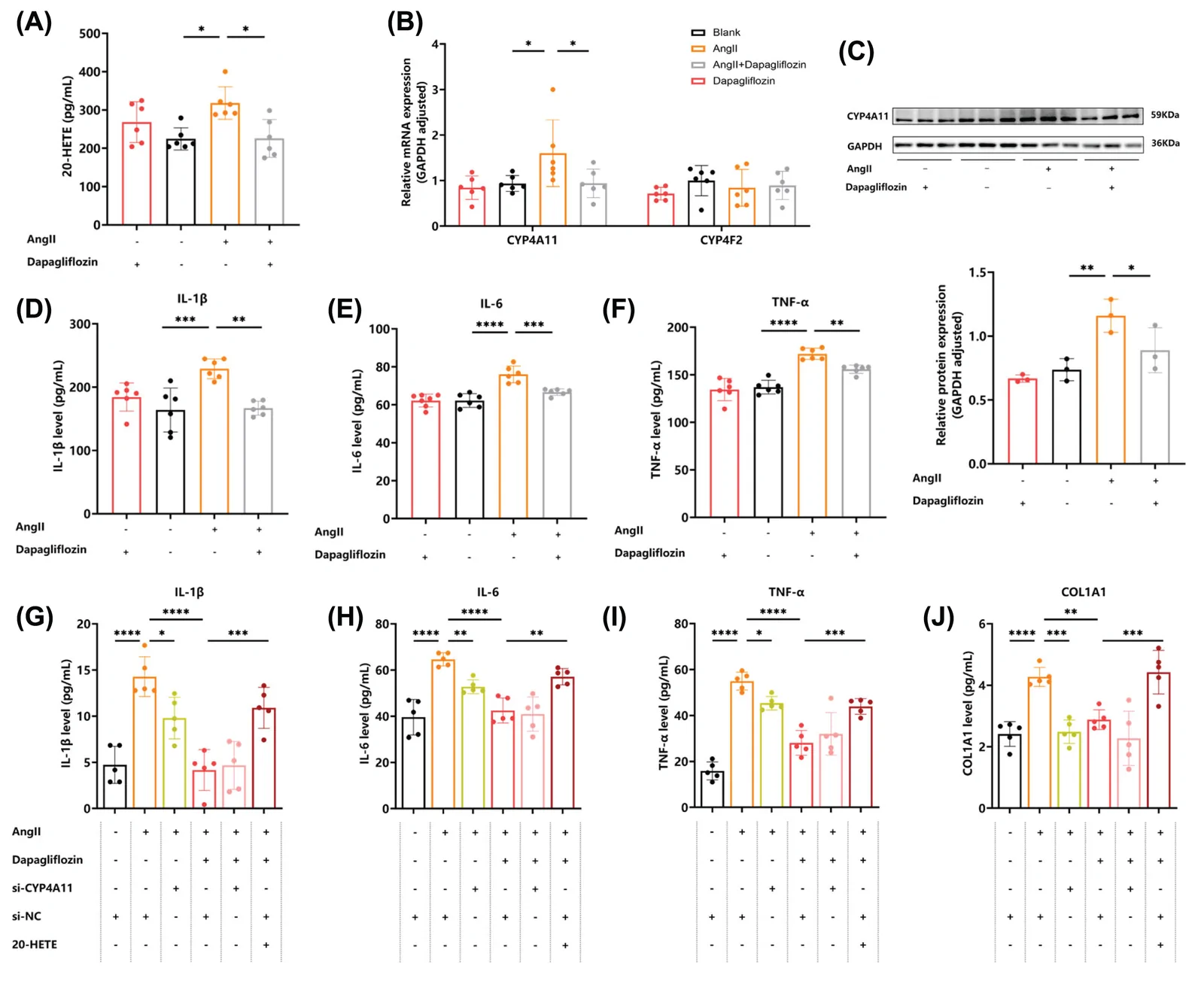
Dapagliflozin ameliorates AngII-induced inflammation and fibrosis in HK2 cells, partially through CYP4A/20-HETE pathway (**A**) The 20-HETE level in the supernatant of HK2 cells among different groups. (**B**) the transcription level of CYP4A11 and CYP4F2 in HK2 among different groups. (**C**) Representative western blot results of CYP4A11 in HK2 of different groups. (**D–J**) Level of IL-1β, IL-6, TNF-α, and COL1A1 in the supernatant of HK2 cells from different groups determined by ELISA. Prespecified pairwise comparisons were conducted by Šídák’s test (normal data) or Dunn’s test (non-normal data). *P<0.05, **P<0.01, ***P<0.001.

To validate our *in vivo* findings, we performed cellular experiments using human renal cortical proximal tubular epithelial cells (HK2) stimulated with AngII [30]. Following dosage optimization (Supplementary Figures S4 and S5), 1 μM AngII and 2.5 μM dapagliflozin were selected for subsequent intervention. AngII significantly increased 20-HETE production (adjusted *P* = 0.025), and this effect was partially reversed by dapagliflozin (adjusted *P* = 0.019) (Figure 4A). No significant difference in 20-HETE levels was observed between the blank group and dapagliflozin-only group (adjusted *P* = 0.307).

Transcriptional changes were predominantly observed in CYP4A11 rather than CYP4F2 (Figure 4B). AngII significantly up-regulated CYP4A11 mRNA expression in HK2 cells (adjusted *P* = 0.042), and this up-regulation was effectively suppressed by dapagliflozin (adjusted *P* = 0.043). Western blot analysis (Figure 4C) confirmed that dapagliflozin attenuated the AngII-induced increase in CYP4A11 protein expression, consistent with the RT-PCR results. These findings indicate that dapagliflozin reduces AngII-stimulated 20-HETE synthesis by inhibiting CYP4A11 transcription and translation in HK2 cells. Importantly, similar results were also obtained with other SGLT2 inhibitors (empagliflozin and canagliflozin), suggesting that the observed effects are not specific to dapagliflozin (Supplementary Figure S6).

Furthermore, measurement of inflammatory markers (IL-1β, IL-6, and TNF-α) in the cell supernatants and COL1A1 expression in the cells (Figure 4D–F and Supplementary Figure S3F,G) demonstrated that dapagliflozin suppressed AngII-induced inflammation and fibrotic responses in HK2 cells, consistent with the results of animal experiments. Given the observed anti-inflammatory and antifibrotic effects of dapagliflozin, we next performed loss-of-function and rescue experiments to validate the underlying mechanism (Supplementary Figure S7). siRNA-mediated knockdown of CYP4A11 significantly attenuated AngII-induced elevations in the supernatant levels of IL-1β, IL-6, and TNF-α, as well as COL1A1 expression in HK2 cells (Figure 4G–J). Conversely, supplementation with exogenous 20-HETE effectively reversed the suppressive effects of dapagliflozin on these inflammatory cytokines and COL1A1 levels. These findings provide a close link between CYP4A/20-HETE down-regulation and the anti-inflammatory and antifibrotic effects of dapagliflozin.

## Discussion

This study explored the antihypertensive effects of SGLT2i through a retrospective cohort study and validated these findings in an SHR model. Our results not only confirmed the blood pressure-lowering effect of SGLT2i in hypertension but also suggested that this effect is partially mediated by the renal CYP4A/20-HETE pathway.

Gliflozin, a newer class of antidiabetic agents, has demonstrated benefits in blood pressure management since its introduction for treating type 2 diabetes mellitus. For example, the EMPA-REG OUTCOME trial, which included 825 patients with type 2 diabetes mellitus and hypertension, revealed significant reductions in 24-h blood SBP (1.4–6.6 mmHg) and mean blood pressure (0.4–2.5 mmHg) with empagliflozin treatment [31]. Similar blood pressure-lowering effects of dapagliflozin and canagliflozin [32] have also been observed in patients with type 2 diabetes and hypertension [9]. However, while these trials demonstrated the antihypertensive effects of SGLT2i in diabetic populations, studies specifically investigating their efficacy in hypertensive patients—regardless of diabetic status—remain limited. To address this gap, our retrospective study enrolled a cohort of hypertensive patients irrespective of diabetic history. This design allowed us to adjust for the potential confounding effect of diabetes on the antihypertensive efficacy of SGLT2i. After adjusting for baseline comorbidities and conventional antihypertensive therapies via generalized linear model analysis and PSM, SGLT2i treatment remained significantly associated with reductions in both systolic and diastolic blood pressure. These findings demonstrate that the antihypertensive effect of SGLT2i is independent of concomitant blood-pressure-lowering medications and comorbid conditions (Figure 1 and Table 1). These results were further validated in our SHR animal model (Figure 2) and are consistent with previous reports using other hypertensive models [33,34], reinforcing the therapeutic potential of SGLT2i for managing hypertension independent of diabetic status.

Notably, although SGLT2i treatment was initiated at 8 weeks of age, a significant reduction in blood pressure did not emerge until 28 weeks of age, suggesting the involvement of chronic mechanisms rather than an immediate hemodynamic effect. This delayed onset may be partly explained by the lower expression of SGLT2 in the proximal tubule of SHRs compared with diabetic models [35], which may limit the magnitude of osmotic diuresis. Despite this delayed onset, long-term blood pressure monitoring revealed a sustained antihypertensive effect. Given that the plasma volume reduction induced by SGLT2i is transient [36], these findings further indicate that alleviation of chronic inflammation and fibrosis—beyond natriuresis and glucose-associated osmotic diuresis—contribute to the sustained blood pressure-lowering effects (Figures 3 and 4).

AA metabolites—including EETs/DHETs, HETEs, and prostaglandins—are key regulators of blood pressure. In our study, SHRs exhibited elevated levels of DHETs and HETEs, further supporting the involvement of AA metabolism in hypertension (Supplementary Table S5). In addition, multi-omics studies have linked gliflozin to altered AA metabolism in diabetic cardiomyopathy [37] and non-alcoholic fatty liver disease [38]. We found that 20-week SGLT2i treatment attenuated blood pressure progression in SHRs, accompanied by reduced plasma 20-HETE. Although CYP4A enzymes—which catalyze 20-HETE synthesis—are widely distributed across tissues, SGLT2i-mediated suppression of 20-HETE occurred primarily in the renal cortex but not in the heart or aorta (Figure 3). Immunofluorescence and cellular experiments (Figure 4) confirmed that SGLT2i inhibit CYP4A transcription and translation in proximal tubular epithelial cells, thereby reducing 20-HETE synthesis. These findings suggest that SGLT2i regulates blood pressure partly through the renal CYP4A/20-HETE pathway.

The association between the CYP4A/20-HETE axis and hypertension has been well documented. For example, genetic variants in 20-HETE-producing CYP enzymes are associated with hypertension [39], and the identification of dentification of GPR75 as the 20-HETE receptor has clarified downstream mechanisms [21]. Through GPR75 activation, 20-HETE promotes angiotensin-converting enzyme expression, vasoconstriction, and endothelial dysfunction [30]. However, the delayed antihypertensive effect of dapagliflozin observed in our SHR model suggests that 20-HETE-mediated vasoconstriction may not be the primary mechanism. Instead, given that 20-HETE promotes renal inflammation [40] and fibrosis [41,42], we propose that lowering 20-HETE ameliorates renal remodeling rather than directly reducing vasoconstriction. This is supported by reduced levels of IL-1β, IL-6, TNF-α, and renal fibrosis *in vivo* and *in vitro* (Figures 3 and 4). Furthermore, CYP4A11 knockdown attenuated AngII-induced inflammation and fibrosis, while exogenous 20-HETE reversed the protective effects of dapagliflozin, confirming a causal role for the CYP4A/20-HETE axis in mediating inflammation and fibrosis induced by dapagliflozin.

Furthermore, our results also demonstrate that CYP4A down-regulation is a common effect shared by multiple SGLT2i (Supplementary Figure S6). This regulation occurs predominantly in the proximal renal tubular epithelium, which is also the primary site of SGLT2 expression. Thus, it is reasonable to hypothesize that this regulation is related to the action of SGLT2i on SGLT2. Recent studies indicate that SGLT2i induce a fasting-like metabolic state by promoting urinary caloric loss via SGLT2, thereby suppressing mTOR and activating the AMPK signaling pathway, which leads to a series of complex downstream modulations [43,44]. Among these, nuclear factor erythroid 2-related factor 2 (NRF2), a downstream effector of the AMPK pathway, has been shown to exert renoprotective effects [45,46]. Although there is no direct evidence, the increase in NRF2 coincides with the decrease in CYP4A in an animal trial treated with SGLT2i [47]. Given their common involvement in oxidative stress, we suspect that a relationship may exist between NRF2 and CYP4A. However, this remains a hypothesis at this stage and warrants further investigation. Moreover, although SGLT2i have also been shown to lower blood pressure through other pathways—modulating TRPC3-mediated vascular calcium handling [34] or the SIRT6/HIF-1α signaling pathway [48] to alleviate high-salt-induced renal injury—we propose that SGLT2i exert antihypertensive effects, at least in part, by inhibiting the CYP4A/20-HETE pathway, which may broaden their clinical applications.

Although CYP inhibitors and 20-HETE receptor blockers have been explored preclinically, certain limitations restrict their application. For example, dibromo-dodecenyl-methylsulfimide, a selective inhibitor of 20-HETE synthesis, also affects the production of EETs, leading to potential side effects [39]. Similarly, while N-disodium succinate-20-hydroxyeicosa-6 (Z), 15(Z)-diencarboxamide has been shown to block the 20-HETE effect in animal models [49], the absence of clinical trials limits its broader use. Given their established safety profile, SGLT2i offer a promising alternative for targeting this pathway. To further explore the interaction between SGLT2i and CYP4A, we conducted molecular docking simulations, which predicted that dapagliflozin binds effectively to CYP4A, with a calculated binding affinity of −8.8 kcal/mol (Supplementary Figure S8). All tested SGLT2i showed low binding energies, suggesting stable interactions pending further validation.

In addition, it should be noted that the hypertensive animal model used in our study is also characterized by sympathetic nerve overactivation [50,51]. Chronic sympathetic overactivity is known to contribute to the development of hypertension [52], and the kidney is considered a central hub in this process [53]. In our experiments, dapagliflozin treatment was associated with reduced renal inflammatory cytokines and fibrosis. We propose that the observed decrease in sympathetic tone may be mediated, at least in part, through the alleviation of renal inflammation and fibrosis. These local improvements could, in turn, reduce renal sensory fiber activity, thereby modulating afferent signaling and attenuating central reflex pathways, ultimately contributing to blood pressure reduction [54]. Although we did not directly measure sympathetic nerve activity following treatment, dapagliflozin reduced blood pressure without significantly increasing HR in SHRs, suggesting that SGLT2 inhibition may suppress sympathetic overactivity. This observation aligns with findings from previous animal [33,55] and human studies [53,56], although the mechanism remains unclear.

Several limitations should be acknowledged. First, neither the retrospective study nor the animal experiments demonstrated a persistent antihypertensive effect of SGLT2i on DBP compared with SBP, suggesting that SBP may be more sensitive to SGLT2i—a finding that warrants further investigation. Second, while our findings indicate that SGLT2i lower blood pressure in SHRs through modulation of renal AA metabolism, this represents one of several potential mechanisms underlying their antihypertensive effects. Third, despite adjusting for baseline differences using multiple linear regression and PSM, residual confounding may persist within the cohort and influence the evaluation of actual treatment effects. For example, the restriction of SGLT2i use to patients with diabetes and heart failure may have introduced selection bias. Additionally, we did not assess AA-related metabolites in patients treated with SGLT2i, which should be addressed in future prospective, randomized trials. Finally, hypertension is a multifactorial condition with diverse etiologies, and the use of a single-sex SHR model may not fully recapitulate human hypertension [57]. Moreover, recent studies have suggested that SLGT2i may exert off-target effects in a mouse model of heart failure with reduced ejection fraction [58], highlighting the need for further studies using knockout animal models to confirm whether these antihypertensive effects are independent of SLGT2 inhibition.

## Conclusion

Our study demonstrates that SGLT2i suppress CYP4A transcription and expression in proximal tubular epithelial cells, reducing 20-HETE synthesis and attenuating CYP4A/20-HETE-mediated hypertensive responses. These findings provide a theoretical foundation for repurposing gliflozins as potential therapeutic agents for hypertension management.

## Supplementary Material

Supplementary Figures S1-S8 and Tables S1-S5

## Data Availability

All raw data used in the study are available from the corresponding authors upon reasonable request.

## Abbreviations

AA: arachidonic acid
AngII: Angiotensin II
CON: conventional therapy alone
CYP: cytochrome P450
DHETs: dihydroxy-eicosatetraenoic acids
DBP: Diastolic blood pressure
ELISA: enzyme-linked immunosorbent assay
HR: heart rate
IQR: interquartile range
LC-MS/MS: liquid chromatography–tandem mass spectrometry
NRF2: nuclear factor erythroid 2-related factor 2
20-HETE: 20-hydroxyeicosatetraenoic acid
PSM: propensity score matching
SGLT2i: sodium–glucose transport protein 2 inhibitors
SHRs: spontaneously hypertensive rats
SMD: standardized mean difference
SBP: systolic blood pressure
WGA: Wheat germ agglutinin
WKY: Wistar-Kyoto

## References

[1] Zhou B., Perel P., Mensah G.A. and Ezzati M. (2021) Global epidemiology, health burden and effective interventions for elevated blood pressure and hypertension. Nat. Rev. Cardiol. 18, 785–802 34050340 10.1038/s41569-021-00559-8 PMC8162166

[2] Olsen M.H., Angell S.Y., Asma S., Boutouyrie P., Burger D., Chirinos J.A. et al. (2016) A call to action and a lifecourse strategy to address the global burden of raised blood pressure on current and future generations: the Lancet Commission on hypertension. Lancet 388, 2665–2712 27671667 10.1016/S0140-6736(16)31134-5

[3] Collaborators G.B.D.R.F. (2018) Global, regional, and national comparative risk assessment of 84 behavioural, environmental and occupational, and metabolic risks or clusters of risks for 195 countries and territories, 1990-2017: a systematic analysis for the Global Burden of Disease Study 2017. Lancet 392, 1923–1994 30496105 10.1016/S0140-6736(18)32225-6 PMC6227755

[4] Collaboration N.C.D.R.F. (2021) Worldwide trends in hypertension prevalence and progress in treatment and control from 1990 to 2019: a pooled analysis of 1201 population-representative studies with 104 million participants. Lancet 398, 957–980 34450083 10.1016/S0140-6736(21)01330-1 PMC8446938

[5] Williams B., Mancia G., Spiering W., Agabiti Rosei E., Azizi M., Burnier M. et al. (2018) 2018 ESC/ESH Guidelines for the management of arterial hypertension. Eur. Heart J. 39, 3021–3104 30165516 10.1093/eurheartj/ehy339

[6] Wiviott S.D., Raz I., Bonaca M.P., Mosenzon O., Kato E.T., Cahn A. et al. (2019) Dapagliflozin and cardiovascular outcomes in type 2 diabetes. N. Engl. J. Med. 380, 347–357 30415602 10.1056/NEJMoa1812389

[7] Zinman B., Wanner C., Lachin J.M., Fitchett D., Bluhmki E., Hantel S. et al. (2015) Empagliflozin, cardiovascular outcomes, and mortality in type 2 diabetes. N. Engl. J. Med. 373, 2117–2128 26378978 10.1056/NEJMoa1504720

[8] Wanner C., Inzucchi S.E., Lachin J.M., Fitchett D., von Eynatten M., Mattheus M. et al. (2016) Empagliflozin and progression of kidney disease in type 2 diabetes. N. Engl. J. Med. 375, 323–334 27299675 10.1056/NEJMoa1515920

[9] Neal B., Perkovic V., Mahaffey K.W., de Zeeuw D., Fulcher G., Erondu N. et al. (2017) Canagliflozin and cardiovascular and renal events in type 2 diabetes. N. Engl. J. Med. 377, 644–657 28605608 10.1056/NEJMoa1611925

[10] McMurray J.J.V., Solomon S.D., Inzucchi S.E., Kober L., Kosiborod M.N., Martinez F.A. et al. (2019) Dapagliflozin in patients with heart failure and reduced ejection fraction. N. Engl. J. Med. 381, 1995–2008 31535829 10.1056/NEJMoa1911303

[11] Packer M., Anker S.D., Butler J., Filippatos G., Pocock S.J., Carson P. et al. (2020) Cardiovascular and renal outcomes with empagliflozin in heart failure. N. Engl. J. Med. 383, 1413–1424 32865377 10.1056/NEJMoa2022190

[12] McDonagh T.A., Metra M., Adamo M., Gardner R.S., Baumbach A., Bohm M. et al. (2021) 2021 ESC Guidelines for the diagnosis and treatment of acute and chronic heart failure. Eur. Heart J. 42, 3599–3726 34922348 10.1093/eurheartj/ehab368

[13] Writing Committee M. and Members A.A.J.C. (2022) 2022 AHA/ACC/HFSA Guideline for the management of heart failure. J. Card. Fail. 28, e1–e167 35378257 10.1016/j.cardfail.2022.02.010

[14] Heerspink H.J.L., Stefansson B.V., Correa-Rotter R., Chertow G.M., Greene T., Hou F.F. et al. (2020) Dapagliflozin in patients with chronic kidney disease. N. Engl. J. Med. 383, 1436–1446 32970396 10.1056/NEJMoa2024816

[15] The E.-K.C.G., Herrington W.G., Staplin N., Wanner C., Green J.B., Hauske S.J. et al. (2023) Empagliflozin in patients with chronic kidney disease. N. Engl. J. Med. 388, 117–127 36331190 10.1056/NEJMoa2204233 PMC7614055

[16] Wanner C., Lachin J.M., Inzucchi S.E., Fitchett D., Mattheus M., George J. et al. (2018) Empagliflozin and clinical outcomes in patients with type 2 diabetes mellitus, established cardiovascular disease, and chronic kidney disease. Circulation 137, 119–129 28904068 10.1161/CIRCULATIONAHA.117.028268

[17] Wang T., Fu X., Chen Q., Patra J.K., Wang D., Wang Z. et al. (2019) Arachidonic acid metabolism and kidney inflammation. Int. J. Mol. Sci. 20, 3683 31357612 10.3390/ijms20153683 PMC6695795

[18] Wang B., Wu L., Chen J., Dong L., Chen C., Wen Z. et al. (2021) Metabolism pathways of arachidonic acids: mechanisms and potential therapeutic targets. Signal Transduct. Target Ther. 6, 94 33637672 10.1038/s41392-020-00443-w PMC7910446

[19] Zhou Y., Khan H., Xiao J. and Cheang W.S. (2021) Effects of arachidonic acid metabolites on cardiovascular health and disease. Int. J. Mol. Sci. 22, 12029 34769460 10.3390/ijms222112029 PMC8584625

[20] Imig J.D. (2019) Epoxyeicosanoids in hypertension. Physiol. Res. 68, 695–704 31475560 10.33549/physiolres.934291 PMC6941753

[21] Garcia V., Gilani A., Shkolnik B., Pandey V., Zhang F.F., Dakarapu R. et al. (2017) 20-HETE signals through G-protein-coupled receptor GPR75 (G(q)) to affect vascular function and trigger hypertension. Circ. Res. 120, 1776–1788 28325781 10.1161/CIRCRESAHA.116.310525 PMC5446268

[22] Roman R.J. (2024) 20-HETE and hypertension. Hypertension 81, 2012–2015 39193710 10.1161/HYPERTENSIONAHA.124.21718 PMC11410503

[23] Unger T., Borghi C., Charchar F., Khan N.A., Poulter N.R., Prabhakaran D. et al. (2020) 2020 International Society of Hypertension Global Hypertension Practice Guidelines. Hypertension 75, 1334–1357 32370572 10.1161/HYPERTENSIONAHA.120.15026

[24] Arab H.H., Safar M.M. and Shahin N.N. (2021) Targeting ROS-dependent AKT/GSK-3β/NF-κB and DJ-1/Nrf2 pathways by dapagliflozin attenuates neuronal injury and motor dysfunction in rotenone-induced Parkinson’s disease rat model. ACS Chem. Neurosci. 12, 689–703 33543924 10.1021/acschemneuro.0c00722

[25] Tang Y., Tan S., Li M., Tang Y., Xu X., Zhang Q. et al. (2022) Dapagliflozin, sildenafil and their combination in monocrotaline-induced pulmonary arterial hypertension. BMC Pulm. Med. 22, 142 35413880 10.1186/s12890-022-01939-7 PMC9006601

[26] Wojcikowski K. and Gobe G. (2014) Animal studies on medicinal herbs: predictability, dose conversion and potential value. Phytother. Res. 28, 22–27 23553964 10.1002/ptr.4966

[27] Zhang X., Yang N., Ai D. and Zhu Y. (2015) Systematic metabolomic analysis of eicosanoids after omega-3 polyunsaturated fatty acid supplementation by a highly specific liquid chromatography-tandem mass spectrometry-based method. J. Proteome Res. 14, 1843–1853 25736083 10.1021/pr501200u

[28] Schwartzman M.L., Lin F., Nishimura M. and Abraham N.G. (1996) Cytochrome P450 4A expression and arachidonic acid omega-hydroxylation in the kidney of the spontaneously hypertensive rat. Nephron 73, 652–663 10.1159/000189154 8856265

[29] Kroetz D.L. and Xu F. (2005) Regulation and inhibition of arachidonic acid omega-hydroxylases and 20-HETE formation. Annu. Rev. Pharmacol. Toxicol. 45, 413–438 15822183 10.1146/annurev.pharmtox.45.120403.100045

[30] Rocic P. and Schwartzman M.L. (2018) 20-HETE in the regulation of vascular and cardiac function. Pharmacol. Ther. 192, 74–87 30048707 10.1016/j.pharmthera.2018.07.004 PMC6278600

[31] Tikkanen I., Narko K., Zeller C., Green A., Salsali A., Broedl U.C. et al. (2015) Empagliflozin reduces blood pressure in patients with type 2 diabetes and hypertension. Diabetes Care 38, 420–428 25271206 10.2337/dc14-1096

[32] Pfeifer M., Townsend R.R., Davies M.J., Vijapurkar U. and Ren J. (2017) Effects of canagliflozin, a sodium glucose co-transporter 2 inhibitor, on blood pressure and markers of arterial stiffness in patients with type 2 diabetes mellitus: a post hoc analysis. Cardiovasc. Diabetol. 16, 29 28241822 10.1186/s12933-017-0511-0 PMC5327537

[33] Herat L.Y., Magno A.L., Rudnicka C., Hricova J., Carnagarin R., Ward N.C. et al. (2020) SGLT2 inhibitor-induced sympathoinhibition: a novel mechanism for cardiorenal protection. JACC Basic Transl. Sci. 5, 169–179 32140623 10.1016/j.jacbts.2019.11.007 PMC7046513

[34] Zhao Y., Li L., Lu Z., Hu Y., Zhang H., Sun F. et al. (2022) Sodium-glucose cotransporter 2 inhibitor canagliflozin antagonizes salt-sensitive hypertension through modifying transient receptor potential channels 3 mediated vascular calcium handling. J. Am. Heart Assoc. 11, e025328 35904193 10.1161/JAHA.121.025328 PMC9375510

[35] Gerich J.E. (2010) Role of the kidney in normal glucose homeostasis and in the hyperglycaemia of diabetes mellitus: therapeutic implications. Diabet. Med. 27, 136–142 20546255 10.1111/j.1464-5491.2009.02894.x PMC4232006

[36] Sha S., Polidori D., Heise T., Natarajan J., Farrell K., Wang S.S. et al. (2014) Effect of the sodium glucose co-transporter 2 inhibitor canagliflozin on plasma volume in patients with type 2 diabetes mellitus. Diabetes Obes. Meta. 16, 1087–1095 24939043 10.1111/dom.12322

[37] Xi Y., Chen D., Dong Z., Zhang J., Lam H., He J. et al. (2022) Multi-omics insights into potential mechanism of SGLT2 inhibitors cardiovascular benefit in diabetic cardiomyopathy. Front. Cardiovasc. Med. 9, 999254 10.3389/fcvm.2022.999254 PMC9579694 36277768

[38] Niu S., Ren Q., Chen S., Pan X., Yue L., Chen X. et al. (2023) Metabolic and hepatic effects of empagliflozin on nonalcoholic fatty liver Mice. Diabetes Metab. Syndr. Obes. 16, 2549–2560 37645238 10.2147/DMSO.S422327 PMC10461752

[39] Froogh G., Garcia V. and Laniado Schwartzman M. (2022) The CYP/20-HETE/GPR75 axis in hypertension. Adv. Pharmacol. 94, 1–25 35659370 10.1016/bs.apha.2022.02.003 PMC10123763

[40] Hoff U., Lukitsch I., Chaykovska L., Ladwig M., Arnold C., Manthati V.L. et al. (2011) Inhibition of 20-HETE synthesis and action protects the kidney from ischemia/reperfusion injury. Kidney Int. 79, 57–65 20962739 10.1038/ki.2010.377 PMC3813968

[41] Zhang C., Booz G.W., Yu Q., He X., Wang S. and Fan F. (2018) Conflicting roles of 20-HETE in hypertension and renal end organ damage. Eur. J. Pharmacol. 833, 190–200 29886242 10.1016/j.ejphar.2018.06.010 PMC6057804

[42] Zhou Z., Fang Q., Li X., Li C. and Huang J. (2026) Arachidonic acid intake promotes hypertension and target-organ fibrosis through CYP4A-mediated 20-HETE overproduction: integrated evidence from human and animal studies. Clin. Exp. Hypertens. 48, 2611130 41491652 10.1080/10641963.2025.2611130

[43] Hoong C.W.S. and Chua M.W.J. (2021) SGLT2 inhibitors as calorie restriction mimetics: insights on longevity pathways and age-related diseases. Endocrinology 162, bqab079 33857309 10.1210/endocr/bqab079

[44] Gao Y.M., Feng S.T., Wen Y., Tang T.T., Wang B. and Liu B.C. (2022) Cardiorenal protection of SGLT2 inhibitors—perspectives from metabolic reprogramming. eBioMedicine 83, 104215 35973390 10.1016/j.ebiom.2022.104215 PMC9396537

[45] Chen X., Yan Z. and Wang M. (2026) Advances in targeted treatment of ferroptosis in diabetic kidney disease with SGLT2 inhibitors. Diabetol. Metab. Syndr. 18, 163 42216164 10.1186/s13098-026-02193-1 PMC13404099

[46] Lu Q., Yang L., Xiao J.J., Liu Q., Ni L., Hu J.W. et al. (2023) Empagliflozin attenuates the renal tubular ferroptosis in diabetic kidney disease through AMPK/NRF2 pathway. Free Radic. Biol. Med. 195, 89–102 10.1016/j.freeradbiomed.2022.12.088 36581059

[47] Hüttl M., Markova I., Miklankova D., Zapletalova I., Poruba M., Haluzik M. et al. (2021) In a prediabetic model, empagliflozin improves hepatic lipid metabolism independently of obesity and before onset of hyperglycemia. Int. J. Mol. Sci. 22, 11513 34768942 10.3390/ijms222111513 PMC8584090

[48] Wang Q., Liang Y., Zuo Q., He L., Zhang T., Wang Z. et al. (2025) Canagliflozin ameliorates high-salt-induced renal injury and premature aging in male Dahl salt-sensitive rats, with associated changes in SIRT6/HIF-1α signaling. Ren. Fail. 47, 2546624 40796808 10.1080/0886022X.2025.2546624 PMC12344669

[49] Gilani A., Pandey V., Garcia V., Agostinucci K., Singh S.P., Schragenheim J. et al. (2018) High-fat diet-induced obesity and insulin resistance in CYP4a14(-/-) mice is mediated by 20-HETE. Am. J. Physiol. Regul. Integr. Comp. Physiol. 315, R934–R944 30088983 10.1152/ajpregu.00125.2018 PMC6295494

[50] Lundin S., Ricksten S.E. and Thorén P. (1984) Renal sympathetic activity in spontaneously hypertensive rats and normotensive controls, as studied by three different methods. Acta Physiol. Scand. 120, 265–272 6711341 10.1111/j.1748-1716.1984.tb00133.x

[51] Simms A.E., Paton J.F., Pickering A.E. and Allen A.M. (2009) Amplified respiratory-sympathetic coupling in the spontaneously hypertensive rat: does it contribute to hypertension? J. Physiol. 587, 597–610 19064613 10.1113/jphysiol.2008.165902 PMC2670083

[52] Grassi G. (1998) Role of the sympathetic nervous system in human hypertension. J. Hypertens. 16, 1979–1987 9886886 10.1097/00004872-199816121-00019

[53] Sano M. (2018) A new class of drugs for heart failure: SGLT2 inhibitors reduce sympathetic overactivity. J. Cardiol. 71, 471–476 29415819 10.1016/j.jjcc.2017.12.004

[54] Katsurada K. (2025) Interaction between SGLT2 and the sympathetic nervous system in normal and various cardiovascular metabolic disease states. Hypertens Res. 48, 2072–2078 40316758 10.1038/s41440-025-02216-w

[55] Kim H.K., Ishizawa R., Fukazawa A., Wang Z., Bezan Petric U., Hu M.C. et al. (2022) Dapagliflozin attenuates sympathetic and pressor responses to stress in young prehypertensive spontaneously hypertensive rats. Hypertension 79, 1824–1834 35652337 10.1161/HYPERTENSIONAHA.122.19177 PMC9308730

[56] Jordan J., Tank J., Heusser K., Heise T., Wanner C., Heer M. et al. (2017) The effect of empagliflozin on muscle sympathetic nerve activity in patients with type II diabetes mellitus. J. Am. Soc. Hypertension 11, 604–612 10.1016/j.jash.2017.07.005 28757109

[57] Lerman L.O., Kurtz T.W., Touyz R.M., Ellison D.H., Chade A.R., Crowley S.D. et al. (2019) Animal models of hypertension: a scientific statement from the American Heart Association. Hypertension 73, e87–e120 30866654 10.1161/HYP.0000000000000090 PMC6740245

[58] Dyck J.R.B., Sossalla S., Hamdani N., Coronel R., Weber N.C., Light P.E. et al. (2022) Cardiac mechanisms of the beneficial effects of SGLT2 inhibitors in heart failure: evidence for potential off-target effects. J. Mol. Cell Cardiol. 167, 17–31 35331696 10.1016/j.yjmcc.2022.03.005

