# Supplementary material for: SGLT2 inhibitor ameliorates hypertension by regulating the CYP4A/20-HETE pathway in the kidney
Source: Biosci Rep. 2026 Sep 17;46(10):BSR20260150. doi: 10.1042/BSR20260150 (PMC13591247; doi:10.1042/BSR20260150)

Supplemental information

SGLT2 inhibitor ameliorates hypertension by regulating the CYP4A/20-HETE pathway in the kidney

Zhou ZT et al

Supplementary Tables:

Table S1: Primers sequence used in this study

Table S2: Baseline characteristics of the conventional therapy and conventional therapy plus gliflzoins group

Table S3: Generalized linear models of systolic blood pressure in hypertensive population after kinds of treatment

Table S4: Generalized linear models of diastolic blood pressure in hypertensive population after kinds of treatment

Table S5: Plasma concentrations of arachidonic acid related metabolites in SHR rats with or without gliflozin treatment.

Supplemental Figures:

Figure S1. Antihypertensive effect of different SGLT2 inhibitors.

Figure S2. Dapagliflozin ameliorates cardiac fibrosis in SHRs.

Figure S3. Dapagliflozin ameliorates renal fibrosis in SHRs.

Figure S4. 20-HETE concentration in culture medium of HK2 cells following AngII treatment.

Figure S5. Dose-dependent effects of dapagliflozin on CYP4A11 expression in AngII-stimulated HK-2 cells.

Figure S6. Empagliflozin and canagliflozin attenuate AngII-induced 20-HETE production in HK2 cells.

Figure S7. Knockdown efficiency of si-CYP4A.

Figure S8. Molecular docking results of CYP4A with various SGLT2 inhibitors.

Table S1 Primers sequence used in this study

Primer	Sequence（5'-3')
r-CYP4A1-F[1]	CTCTTACTTGCCAGAATGGAGAA
r-CYP4A1-R[1]	GACTTGGATACCCTTGGGTAAAG
r-CYP4A2-F[1]	AGATCCAAAGCCTTATCAATC
r-CYP4A2-R[1]	CAGCCTTGGTGTAGGACCT
r-CYP4A3-F[1]	CAAAGGCTTCTGGAATTTATC
r-CYP4A3-R[1]	CAGCCTTGGTGTAGGACCT
r-CYP4F1&4-F	TGCTGACACCTGCCTTCCAC
r-CYP4F1&4-R	TTGGCGTGCATGATGTTGGT
r-GAPDH-F	TATGACTCTACCCACGGCAAG
r-GAPDH-R	ATACTCAGCACCAGCATCACC
h-CYP4A11-F	AAGACATTCCCAAGTGCCTGT
h-CYP4A11-R	CTGATCTCCCCAGAATCACCT
h-CYP4F2-F	GGTCATCTCCCGCCATGTCA
h-CYP4F2-R	CCACACAGCTGGGTTGTGAT
h-GAPDH-F	CAAATTCCATGGCACCGTCA
h-GAPDH-R	GATGGCATGGACTGTGGTCA

Table S2 Baseline characteristics of the conventional therapy and conventional therapy plus gliflzoins group

	conventional therapy N=364	conventional plus gliflozins N=582	SMD	*P* value
**Basic information**				
Age, years	60 (51 - 69)	60 (53 - 68)	0.015	0.747
Sex, male (%)	235 (64.6)	404 (69.4)	0.102	0.121
BMI (Kg/m^2)	24.4 (22.3 - 26.5)	25.3 (23.3 - 27.6)	**0.261**	**< 0.001**
Smoking (%)	91 (25.0)	215 (36.9)	**0.260**	**＜0.001**
Drinking (%)	52 (14.3)	130 (22.3)	**0.208**	**0.002**
Diabetes (%)	147 (40.4)	573 (98.5)	**1.625**	**＜0.001**
CAD (%)	165 (45.3)	270 (46.4)	0.022	0.750
Cardiomyopathy (%)	22 (6.0)	19 (3.3)	**-0.128**	**0.041**
Arrhythmia (%)	53 (14.6)	60 (10.3)	-0.131	0.050
**Laboratory results**				
HbA1c (%)	5.90 (5.53 - 6.80)	7.80 (6.80 - 9.23)	**1.012**	**＜0.001**
LDL-C (mmol/L)	2.14 (1.64 - 2.91)	2.14 (1.55 - 2.87)	0.007	0.692
HDL-C (mmol/L)	0.98 (0.83 - 1.19)	0.93 (0.80 - 1.10)	**-0.210**	**0.001**
TG (mmol/L)	1.47 (1.01 - 2.34)	1.84 (1.25 - 2.79)	**0.314**	**＜0.001**
eGFR (mL/min/1.73m^2)	83.5 (65.3 - 97.6)	81.8 (62.3 - 97.5)	0.038	0.642
EF (%)	64.0 (59.0 - 67.0)	63.0 (58.0 - 67.0)	-0.063	0.669
**Treatment**				
**Cardiovascular medications (n, %)**				
Alpha-blockers	11 (3.0)	3 (0.5)	**-0.192**	**0.002**
ACEI/ARB	128 (35.2)	238 (40.9)	0.118	0.078
Beta-blockers	139 (38.2)	152 (26.1)	**-0.261**	**＜0.001**
CCB	123 (33.8)	199 (34.2)	0.008	0.899
Diuretics	80 (22.0)	91 (15.6)	**-0.164**	**0.014**
ARNI	4 (1.1)	31 (5.3)	**0.240**	**0.001**
Anticoagulant drugs	35 (9.6)	70 (12.0)	0.077	0.251
Antiplatelet drugs	160 (44.0)	241 (41.4)	-0.053	0.441
Lipid-lowering agents	205 (56.3)	351 (60.3)	0.081	0.225
**Hypoglycemic agents (n, %)**				
SUs	8 (2.2)	50 (8.6)	**0.286**	**＜0.001**
TZDs	5 (1.4)	58 (10.0)	**0.377**	**＜0.001**
Biguanides	35 (9.6)	202 (34.7)	**0.634**	**＜0.001**
DPP-4 inhibitors	29 (8.0)	45 (7.7)	-0.011	0.896
AGIs	65 (17.9)	145 (24.9)	**0.171**	**0.011**
Glinides	3 (0.8)	7 (1.2)	0.040	0.820
GLP-1 agonists	7 (1.9)	80 (13.7)	**0.451**	**＜0.001**
Insulin	69 (19.0)	213 (36.6)	**0.401**	**＜0.001**
**Baseline blood pressure**				
**Initial blood pressure**				
SBP (mmHg)	134 (120 - 146)	137 (123 - 154)	**-**	**0.001**
DBP (mmHg)	78 (70 - 88)	84 (75 - 93)	**-**	**＜0.001**
**Final blood pressure**				
SBP (mmHg)	130 (119 - 143)	128 (118-138)	**-**	**0.020**
DBP (mmHg)	78 (70 – 86)	78 (71 – 85)	-	0.669

**ΔBP**				
ΔSBP	0 (-10 - 2)	-11 (-25 - 2)	**-**	**<0.001**
ΔDBP	0 (-6 - 2)	-6 (-15 - 2)	**-**	**<0.001**

Data were presented as n (%) or median with interquartile range (Q1–Q3).

Keys: BMI, body mass index; CAD, coronary heart disease; HbA1c, glycated hemoglobin/hemoglobin A1c; LDL-C, low-density lipoprotein cholesterol; HDL-C, high density lipoprotein cholesterol; TG, triglyceride; eGFR, estimated glomerular filtration rate; EF, ejection fraction; Alpha blockers, alpha-adrenergic blockers; ACEI, angiotensin converting enzyme inhibitor; ARB, angiotensin receptor blocker; Beta-blockers, beta-adrenergic blockers; CCB, Calcium channel blockers; ARNI, angiotensin receptor-neprilysin inhibitors; SUs, sulfonylureas; TZDs, thiazolidinediones; DPP-4, dipeptidyl peptidase IV; AGIs, Alpha-glucosidase inhibitors; GLP-1, glucagon-like peptide-1; SBP, systolic blood pressure; DBP, diastolic blood pressure. ΔSBP (Final SBP – Initial SBP); ΔDBP (Final DBP – Initial DBP).

Table S3. Generalized linear models of systolic blood pressure change in hypertensive population after kinds of treatment

Dependent variable	Covariate	Univariate		Multivariate	
		β (95%CI)	P	β (95%CI)	P
ΔSBP	**Basic information**				
	Age, years	0.087 (-0.021, 0.194)	0.115		
	Sex, male (%)	1.881 (-0.914, 4.675)	0.187		
	BMI (Kg/m^2)	-0.509 (-0.875, -0.142)	**0.007**	-0.283 (-0.646, 0.079)	0.126
	Smoking (%)	1.757 (-1.040, 4.554)	0.218		
	Drinking (%)	0.953 (-2.369, 4.274)	0.574		
	**Comorbidities-**				
	Diabetes	-7.447 (-10.481, -4.412)	**<0.001**	-2.667 (-7.114, 1.780)	0.240
	CAD	1.049 (-1.577, 3.676)	0.434		
	Cardiomyopathy	2.018 (-4.412, 8.448)	0.539		
	Arrhythmia	0.040 (-3.998, 4.078)	0.985		
	**Laboratory results**				
	HbA1c (%)	-0.787 (-1.441, -0.134)	**0.018**	0.641 (-0.148, 1.429)	0.111
	LDL-C (mmol/L)	-2.024 (-3.353, -0.695)	**0.003**	-1.828 (-3.158, -0.498)	**0.007**
	HDL-C (mmol/L)	-1.138 (-5.787, 3.510)	0.631		
	TG (mmol/L)	-0.478 (-1.285, 0.329)	0.246		
	eGFR, mL/min	-0.041 (-0.093, 0.012)	0.126		
	EF (%)	-0.120 (-0.239, -0.001)	**0.048**	-0.145(-0.271, -0.020)	**0.023**
	**Treatment**				
	**Cardiovascular medications**				
	Alpha-blockers	-6.057 (-16.895, 4.782)	0.273		
	ACEI/ARB	-3.362 (-6.043, -0.682)	**0.014**	-1.320 (-4.225, 1.585)	0.373
	Beta-blockers	-1.588 (-4.424, 1.248)	0.272		
	CCB	-5.166 (-7.911, -2.422)	**<0.001**	-3.202 (-6.071, -0.332)	**0.029**
	Diuretics	-4.110 (-7.503, -0.717)	**0.018**	-4.150 (-7.729, -0.572)	**0.023**
	ARNI	-7.576 (-14.497, -0.655)	**0.032**	-6.218 (-13.587, 1.151)	0.098
	Anticoagulant drugs	-3.278 (-7.442, 0.885)	0.123		
	Antiplatelet drugs	0.957 (-1.692, 3.607)	0.479		
	Hypolipidemic agents	-0.783 (-3.443, 1.877)	0.564		
	**Hypoglycemic agents**				
	Sus	-2.767 (-8.223, 2.689)	0.320		
	**SGLT2i**	-7.926 (-10.570, -5.283)	**<0.001**	-7.098 (-10.755, -3.440)	**<0.001**
	TZDs	-1.691 (-6.942, 3.561)	0.528		
	Biguanides	-4.686 (-7.694, -1.679)	**0.002**	-0.278 (-3.559, 3.002)	0.868
	DPP-4 inhibitors	-3.001 (-7.874, 1.872)	0.227		
	AGIs	-3.593 (-6.736, -0.450)	**0.025**	-1.590 (-4.902, 1.722)	0.347
	Glinides	0.654 (-12.151, 13.459)	0.920		
	GLP-1 agonists	-2.715 (-7.243, 1.813)	0.240		
	Insulin	-0.410 (-3.273, 2.452)	0.779		

Keys: BMI, body mass index; CAD, coronary heart disease; HbA1c, glycated hemoglobin/hemoglobin A1c; LDL-C, low-density lipoprotein cholesterol; HDL-C, high density lipoprotein cholesterol; TG, triglyceride; eGFR, estimated glomerular filtration rate; EF, ejection fraction; Alpha blockers, alpha-adrenergic blockers; ACEI, angiotension converting enzyme inhibitor; ARB, angiotensin receptor blocker; Beta-blockers, beta-adrenergic blockers; CCB, Calcium channel blockers; ARNI, angiotensin receptor-neprilysin inhibitors; SUs, sulfonylureas; TZDs, thiazolidinediones; DPP-4, dipeptidyl peptidase IV; AGIs, Alpha-glucosidase inhibitors; GLP-1, glucagon-like peptide-1; SBP, systolic blood pressure; DBP, diastolic blood pressure. ΔSBP (post-treatment SBP – pre-treatment SBP).

Table S4. Generalized Linear models of diastolic blood pressure change in hypertensive population after kinds of treatment

Dependent variable	Covariate	Univariate		Multivariate	
		β (95%CI)	P	β (95%CI)	P
ΔDBP	**Basic information**				
	Age, years	0.184 (0.115, 0.252)	**<0.001**	0.146 (0.067, 0.226)	**<0.001**
	Sex, male (%)	0.181 (-1.624, 1.986)	0.844		
	BMI (Kg/m^2)	-0.318 (-0.554, -0.081)	**0.009**	-0.082 (-0.322, 0.157)	0.501
	Smoking (%)	1.072 (-0.733, 2.877)	0.244		
	Drinking (%)	0.260 (-1.884, 2.404)	0.812		
	**Comorbidities**				
	Diabetes	-3.310 (-5.281, -1.340)	**0.001**	0.283 (-2.476, 3.042)	0.841
	CAD	2.176 (0.486, 3.866)	**0.012**	0.420 (-1.346, 2.186)	0.641
	Cardiomyopathy	-0.083 (-4.233, 4.068)	0.969		
	Arrhythmia	2.213 (-0.389, 4.815)	0.096		
	**Laboratory results**				
	HbA1c (%)	-0.578 (-0.999, -0.157)	**0.007**	0.348 (-0.161, 0.858)	0.180
	LDL-C (mmol/L)	-1.718 (-2.573, -0.864)	**<0.001**	-1.420 (-2.296, -0.545)	**0.001**
	HDL-C (mmol/L)	0.565 (-2.435, 3.565)	0.712		
	TG (mmol/L)	-0.584 (-1.104, -0.064)	**0.028**	0.092 (-0.442, 0.625)	0.736
	eGFR, mL/min	-0.060 (-0.093, -0.026)	**0.001**	-0.015 (-0.052, 0.022)	0.422
	EF (%)	-0.071 (-0.147, 0.006)	0.072		
	**Treatment**				
	**Cardiovascular medications**				
	Alpha-blockers	-3.836 (-10.831, 3.159)	0.282		
	ACEI/ARB	-2.352 (-4.080, -0.623)	**0.008**	-1.991 (-3.701, -0.281)	**0.023**
	Beta-blockers	-0.658 (-2.489, 1.172)	0.481		
	CCB	-1.389 (-3.170, 0.393)	0.127		
	Diuretics	-1.431 (-3.625, 0.764)	0.201		
	ARNI	-4.043 (-8.513, 0.427)	0.076		
	Anticoagulant drugs	-1.509 (-4.198, 1.180)	0.271		
	Antiplatelet drugs	0.576 (-1.134, 2.286)	0.509		
	Hypolipidemic agents	-0.307 (-2.024, 1.409)	0.726		
	**Hypoglycemic agents**				
	SUs	-2.252 (-5.773, 1.268)	0.210		
	**SGLT2i**	-5.217 (-6.922, -3.512)	**<0.001**	-5.666 (-7.934, -3.398)	**<0.001**
	TZDs	-4.508 (-7.886, -1.130)	**0.009**	-0.334 (-3.988, 3.320)	0.858
	Biguanides	-3.704 (-5.641, -1.768)	**<0.001**	-0.726 (-2.996, 1.543)	0.531
	DPP-4 inhibitors	-1.313 (-4.459, 1.834)	0.414		
	AGIs	-0.481 (-2.515, 1.552)	0.643		
	Glinides	3.645(-4.616, 11.906)	0.387		
	GLP-1 agonists	-1.734 (-4.656, 1.189)	0.245		
	Insulin	-0.602 (-2.450, 1.245)	0.523		

Keys: BMI, body mass index; CAD, coronary heart disease; HbA1c, glycated hemoglobin/hemoglobin A1c; LDL-C, low-density lipoprotein cholesterol; HDL-C, high density lipoprotein cholesterol; TG, triglyceride; eGFR, estimated glomerular filtration rate; EF, ejection fraction; Alpha blockers, alpha-adrenergic blockers; ACEI, angiotension converting enzyme inhibitor; ARB, angiotensin receptor blocker; Beta-blockers, beta-adrenergic blockers; CCB, Calcium channel blockers; ARNI, angiotensin receptor-neprilysin inhibitors; SUs, sulfonylureas; TZDs, thiazolidinediones; DPP-4, dipeptidyl peptidase IV; AGIs, Alpha-glucosidase inhibitors; GLP-1, glucagon-like peptide-1; SBP, systolic blood pressure; DBP, diastolic blood pressure. ΔSBP (post-treatment SBP – pre-treatment SBP);

Table S5. Plasma concentrations of arachidonic acid related metabolites in in SHRs with or without gliflozin treatment.

Metabolites	Groups		
	WKY	SHR	SHR-D
11,12-DHET, pg/mL	3659.9 ± 1111.8	7563.7 ± 2030.6**	7101.9 ± 1374.4*
11,12-EET, ng/mL	91.8 ± 43.3	118.2 ± 40.9	156.8 ± 115.4
12-HETE, ng/mL	213.9 (119.3, 256.1)	706.3 (342.9, 1083.5)*	639.4 (437.1, 1363.4) *
12,13-DiHOME, pg/mL	1385.8 ± 1059.6	5474.9 ± 1575.0**	5778.9 ± 2021.7**
12,13-EpOME, ng/mL	228.2 (119.1, 464.0)	430.9 (316.3, 528.5)	330.1 (303.2, 879.6)
14,15-DHET, pg/mL	293.3 ± 153.7	1033.6 ± 167.4****	1007.3 ± 196.2****
14,15-EET, ng/mL	105.6 ± 47.0	140.1 ± 45.3	173.0 ± 123.3
15-HETE, ng/mL	9.2 (5.0, 16.2)	19.4 (7.8, 33.1)	14.6 (10.7, 38.8)
15-KETE, pg/mL	979.9 (670.6, 4007.6)	653.9 (446.5, 1294.4)	1791.0 (658.7, 3995.0)
15-keto-PGE2, pg/mL	425.1 (337.3, 3103.8)	277.5 (246.2, 822.7)	548.8 (327.1, 1961.1)
17,18-DiHETE, pg/mL	228.3 ± 122.6	161.7 ± 31.3	154.6 ± 45.4
17,18-EpETE, ng/mL	2.3 (1.3, 6.3)	2.9 (1.7, 4.3)	2.1 (2.0, 4.6)
19,20-DiHDPA, pg/mL	192.7 (148.8, 267.2)	345.2 (329.7, 408.4)*	350.7 (271.9, 424.0)
19,20-EpDPA, ng/mL	7.1 (5.1, 12.9)	20.3 (12.5, 23.5)	14.1 (11.9, 31.2)
20-HETE, pg/mL	484.4 ± 124.1	1475.8 ± 371.2***	1050.5 ± 234.8*/#
5-HETE, ng/mL	3.6 (2.5, 5.3)	3.0 (2.3, 4.6)	3.7 (3.1, 5.1)
5-KETE, pg/mL	1302.2 (943.6, 6263.9)	939.6 (680.5, 1742.1)	2184.4 (1005.2, 5335.5)
5,6-DHET, pg/mL	299.6 ± 120.3	543.2 ± 108.7**	627.6 ± 38.4***
5,6-EET, ng/mL	109.8 (96.1, 195.3)	184.3 (118.1, 229.1)	140.3 (120.9, 389.9)
6-keto-PGF1a, pg/mL	147.7 ± 100.0	100.9 ± 77.3	117.2 ± 80.9
8-HETE, ng/mL	1.8 (1.8, 2.9)	4.3 (2.4, 10.2)	6.3 (4.3, 16.9)*
8,9-DHET, pg/mL	101.5 ± 41.5	225.8 ± 44.9**	242.8 ± 53.6***
8,9-EET, ng/mL	56.4 (50.3, 108.9)	97.1 (57.0, 121.6)	75.8 (63.3, 218.9)
9-HODE, ng/mL	6.6 (4.9, 23.2)	11.7 (8.5, 22.6)	12.8 (8.9, 40.1)
AA, ng/mL	2194.2 ± 332.8	3884.8 ± 563.6****	3728.9 ± 234.2***
DHA, ng/mL	1466.6 ± 343.3	4110.0 ± 765.9****	3901.4 ± 825.4***
EPA, ng/mL	226.1 ± 112.7	327.3 ± 80.4	278.5 ± 97.9
LA, mg/mL	12.4 ± 4.3	27.7 ± 5.4***	25.5 ± 3.2**
LXA4, pg/mL	459.4 ± 277.8	383.1 ± 218.0	431.6 ± 386.4
LXB4, pg/mL	613.4 ± 485.1	1569.7 ± 1372.7	750.9 ± 639.0
PGB2, pg/mL	1096.4 ± 833.4	2554.1 ± 1963.2	1334.2 ± 1415.0
PGD2, pg/mL	1617.1 ± 1264.5	3597.1 ± 3210.4	1673.0 ± 1454.7
PGE2, pg/mL	2637.7 ± 1975.5	5395.4 ± 4890.1	3049.7 ± 2942.6
PGF2a, pg/mL	807.0 ± 535.8	1572.4 ± 1254.0	711.9 ± 651.7
TXB2, pg/mL	23712.5 ± 18142.4	55150.4 ± 41053.2	20839.7 ± 15347.8

Data was presented as Mean ± SD or median with interquartile range (Q1–Q3)
WKY vs SHR/ SHR-D: **P*<0.05, ***P*<0.01, ****P*<0.001,
SHR vs SHR-D: #*P*<0.05.
Multiple comparisons were performed using Tukey's HSD test for normally distributed data or Dunn's test for non-normally distributed data

Figure S1. Antihypertensive effect of different SGLT2 inhibitors.

A. The systolic blood pressure change following different types of gliflozins; **B.** the diastolic blood pressure change following different types of gliflozins. CON. Control; DAPA. Dapagliflozin; EMPA. Empagliflozin; CANA. Canagliflozin. **P*<0.05, ***P*<0.01, *****P*<0.0001. Multiple comparisons were performed using Dunn's test.

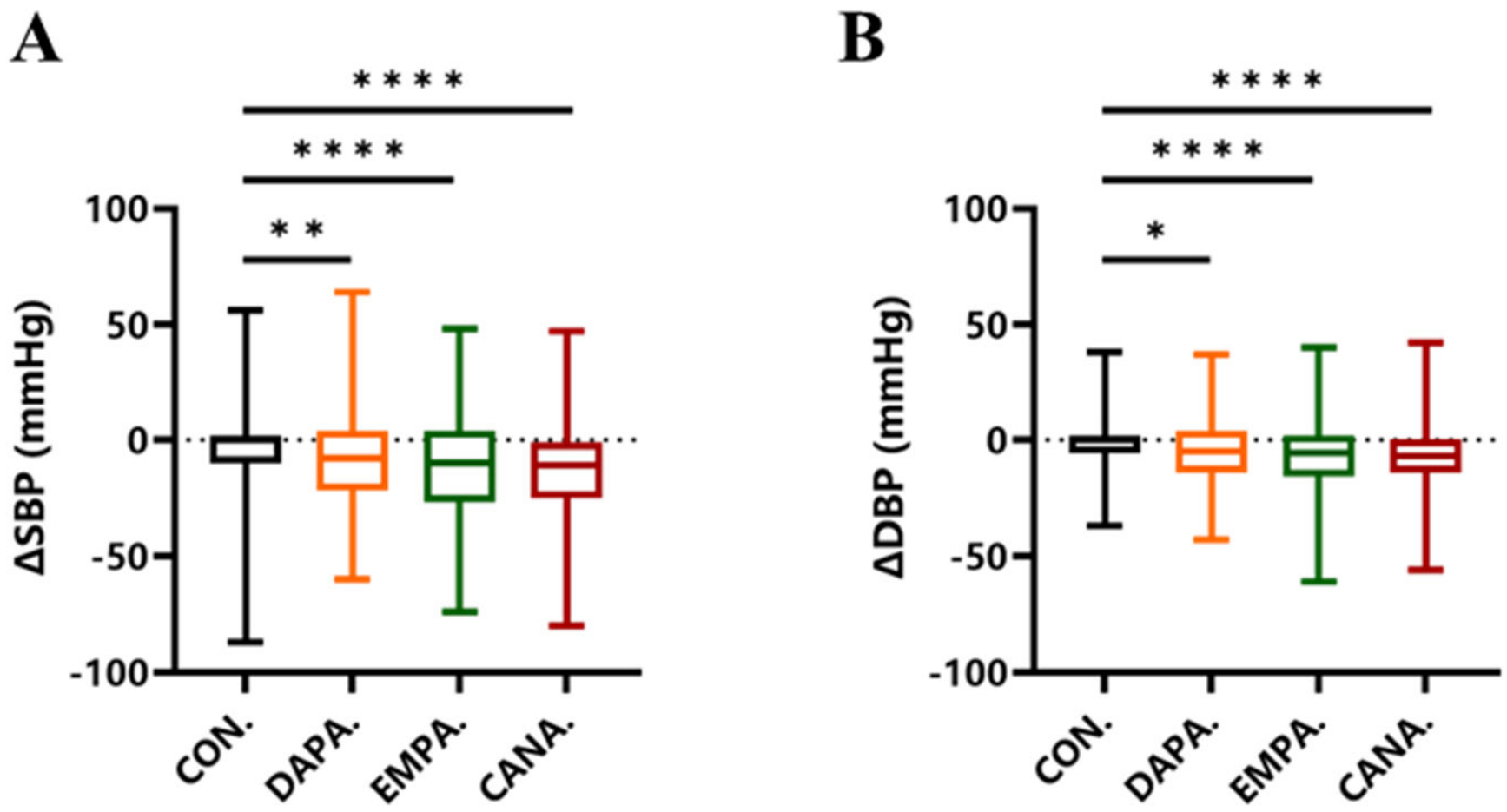

Figure S2. Dapagliflozin ameliorates cardiac fibrosis in SHRs**.**

A, Representative images of wheat germ agglutinin (40×) staining and Masson's trichrome staining (20×) in heart sections; **B**, quantitative analysis of mean cardiomyocyte cross-sectional area; C, quantitative analysis of fibrosis area fraction in heart sections. **P*<0.05, ***P*<0.01, ****P*<0.001.

Multiple comparisons were performed using Tukey's HSD test for normally distributed data or Dunn's test for non-normally distributed data.

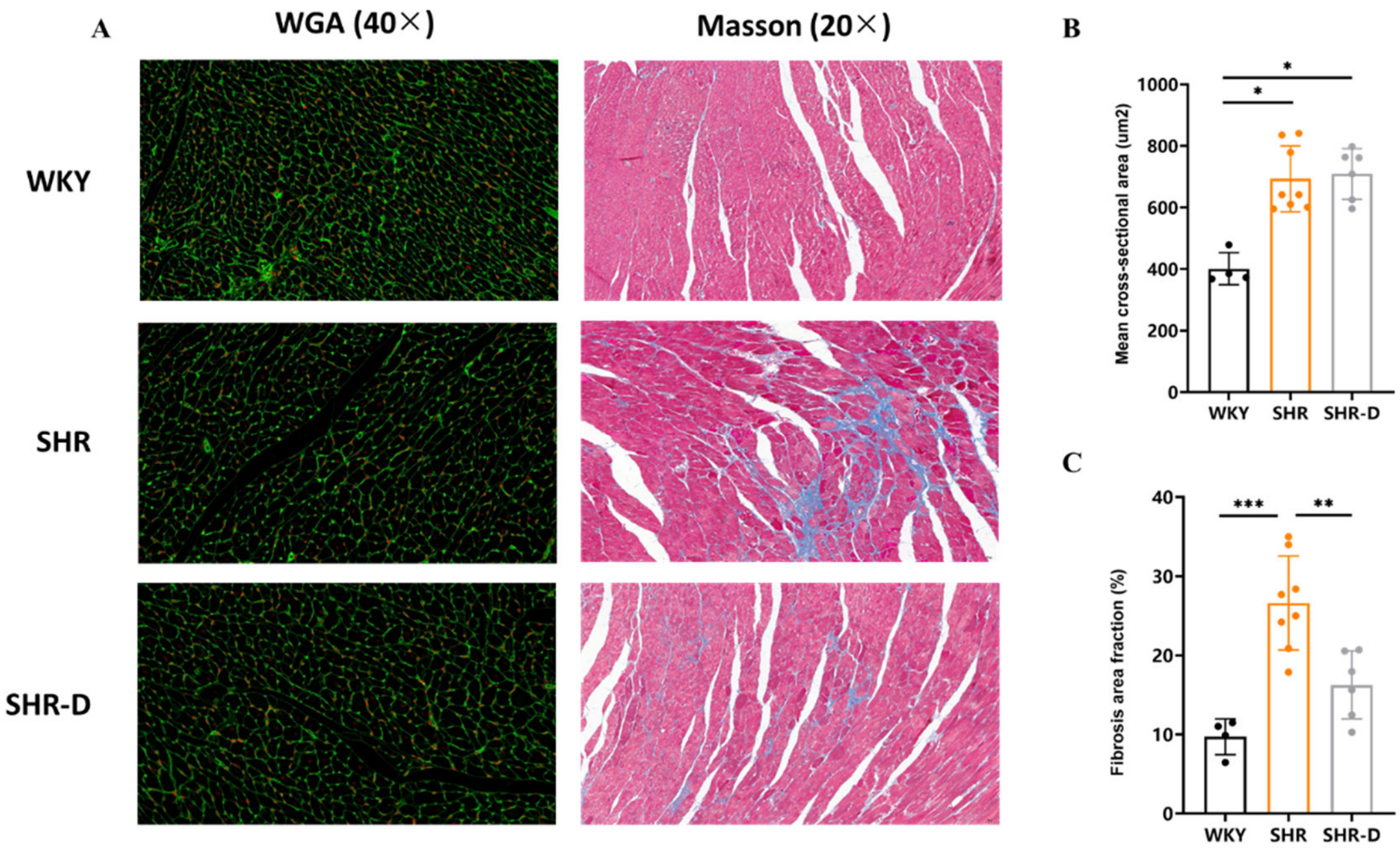

Figure S3. Dapagliflozin ameliorates renal fibrosis in SHRs**.**

A, Representative immunofluorescence staining images of the renal cortex in WKY, SHR, and SHR-D groups; **B**, **C**, Quantitative analysis of mean fluorescence intensity of NHE3 and CYP4A; **D**, Representative Masson's trichrome staining (20×) of the renal cortex; **E**, Quantitative analysis of fibrotic area fraction in the renal cortex; **F**, Representative western blot results of COL1A1 and NHE3 in HK2 cells across experimental groups; **G**, **H**, Quantitative analysis of relative expression levels (normalized to GAPDH) of COL1A1 and NHE3.

*P < 0.05, **P < 0.01, ***P < 0.001, ****P < 0.0001.

B–E: Tukey's HSD test (normal data) or Dunn's test (non-normal data) for multiple comparisons.

G–H: Pre-specified pairwise comparisons (Blank vs AngII, AngII vs AngII+Dapagliflozin, Blank vs Dapagliflozin): Šídák's test (normal) or Dunn's test (non-normal).

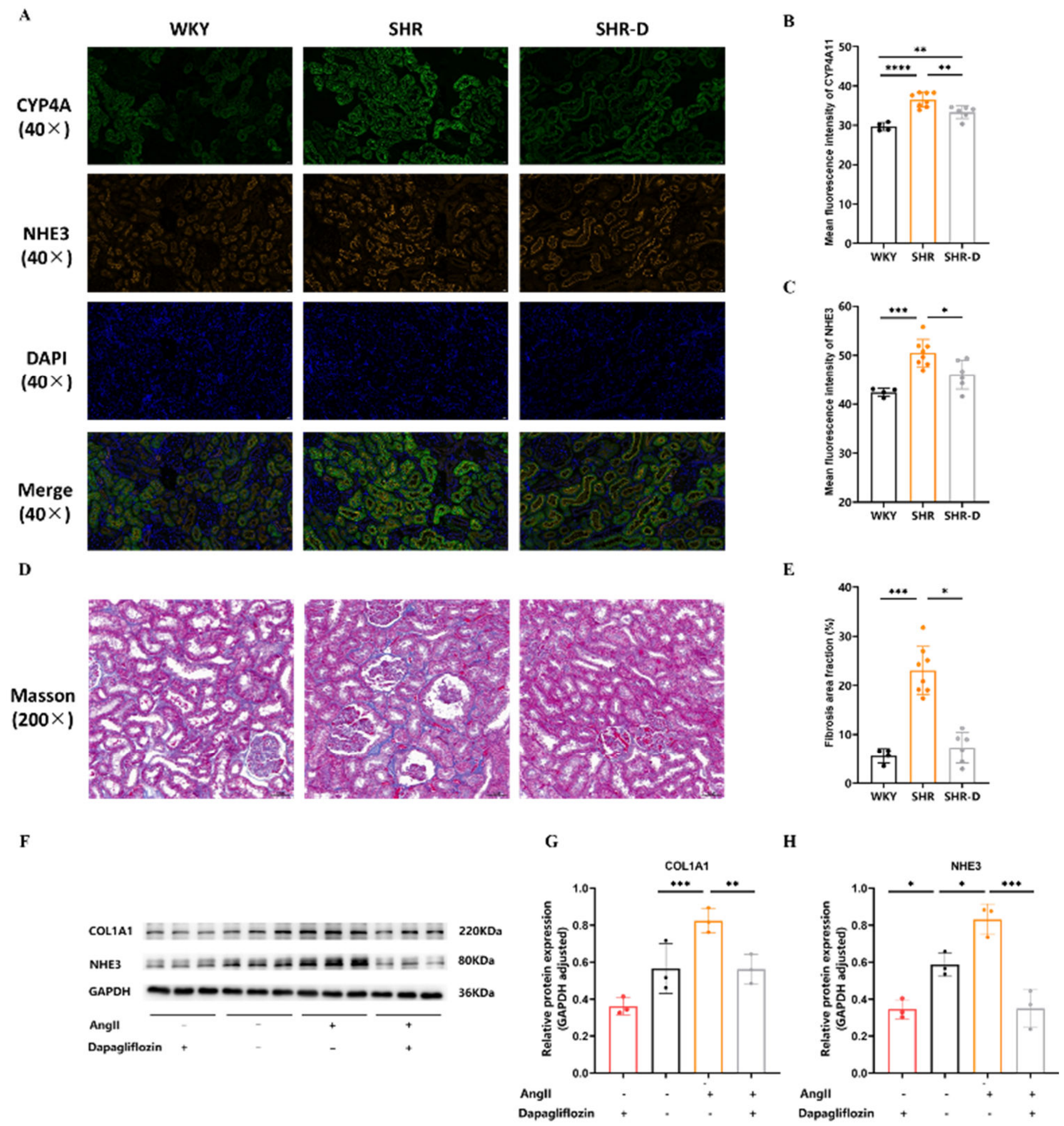

Figure S4. 20-HETE concentration in culture medium of HK2 cells following AngII treatment. *P<0.05.

Pre-specified pairwise comparisons (0 vs 1-100) were performed using Šídák's test.

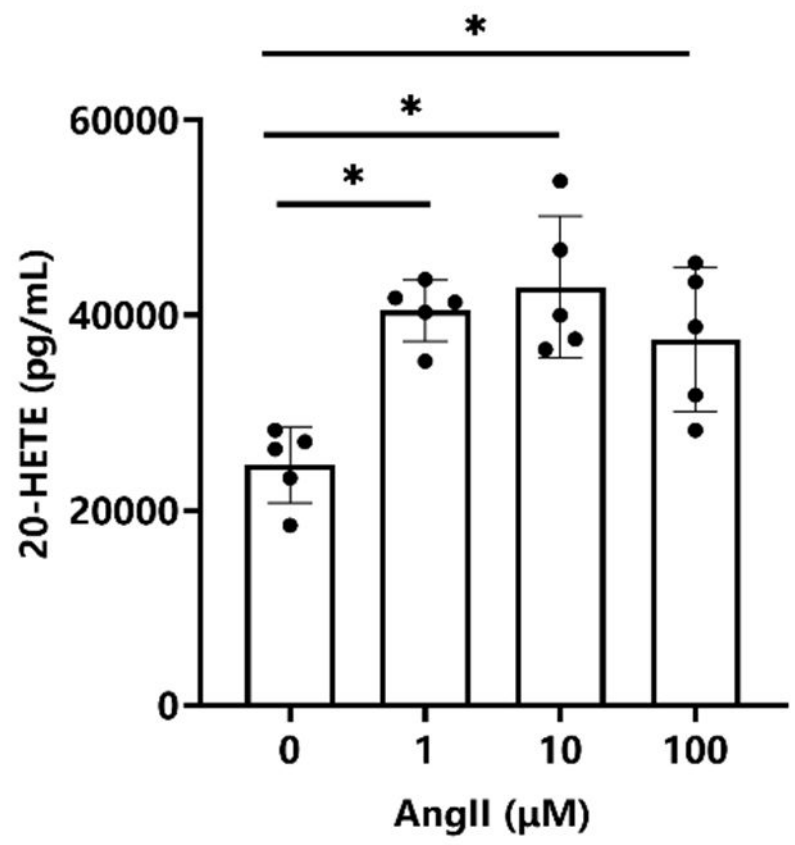

Figure S5. Dose-dependent effects of dapagliflozin on CYP4A11 expression in AngII-stimulated HK-2 cells.

(A) Representative western blot images of CYP4A11 protein expression. (B) Densitometric quantification of CYP4A11 levels normalized to GAPDH.

*P < 0.05, **P < 0.01, ****P < 0.0001. Pre-specified pairwise comparisons (Blank vs. AngII; AngII vs. AngII + dapagliflozin at each indicated concentration) were analyzed using Šídák's multiple comparisons test. Data are shown as mean ± SD.

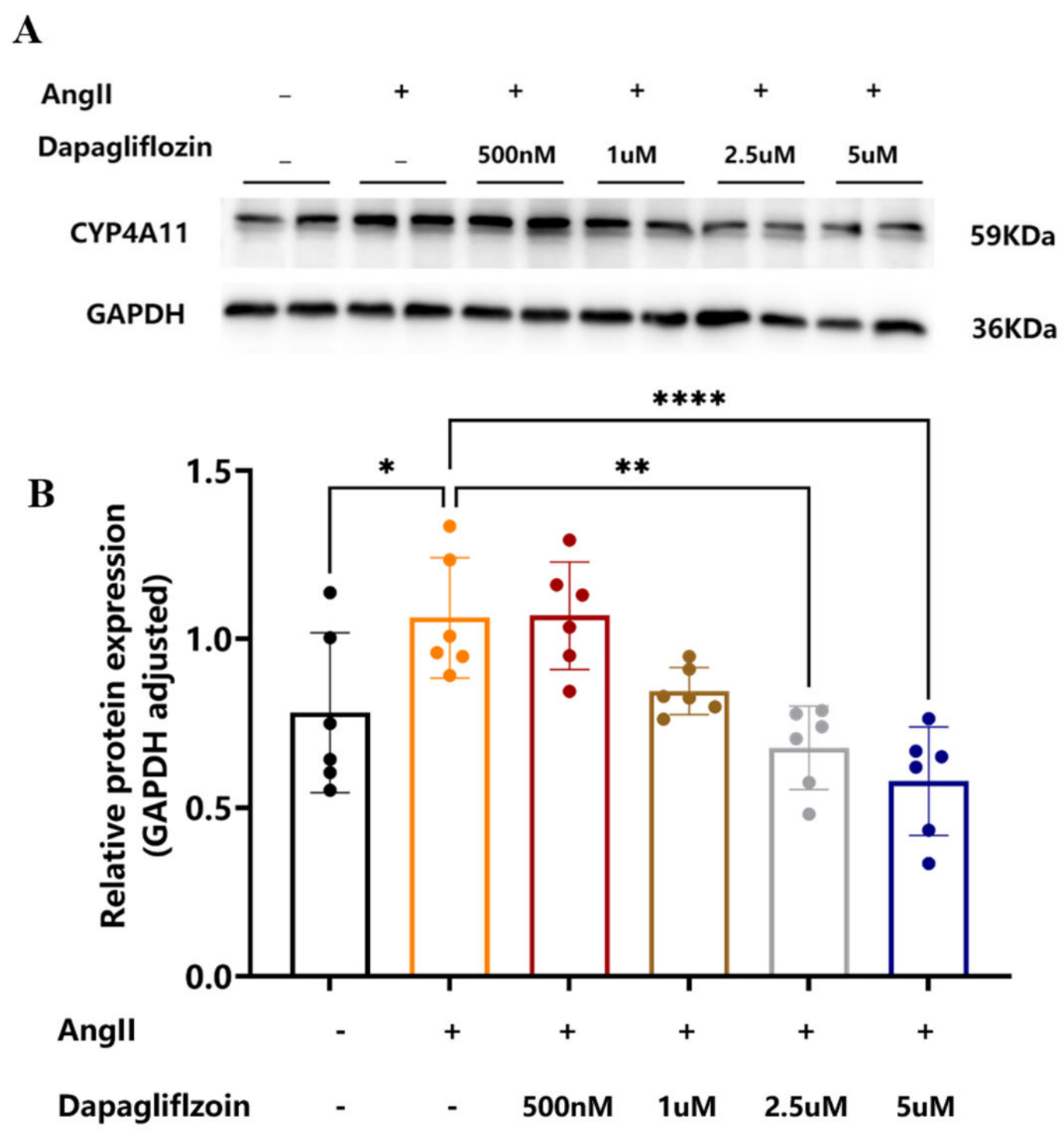

Figure S6. Empagliflozin and canagliflozin attenuate AngII-induced 20-HETE production in HK2 cells.

A, **B**, CYP4A11 protein expression (Western blot and quantification) after AngII treatment with increasing empagliflozin or canagliflozin. **C**, **D**, CYP4A11 mRNA levels after AngII plus empagliflozin or canagliflozin at 5 μM. **E**, Supernatant 20-HETE levels under the same conditions.

*P < 0.05, **P < 0.01, ***P < 0.001, ****P < 0.0001.

Pre-specified comparisons: Blank vs. AngII; AngII vs. AngII + drug (empagliflozin: 500 nM–10 μM; canagliflozin: 1 μM–20 μM) by Šidák's test.

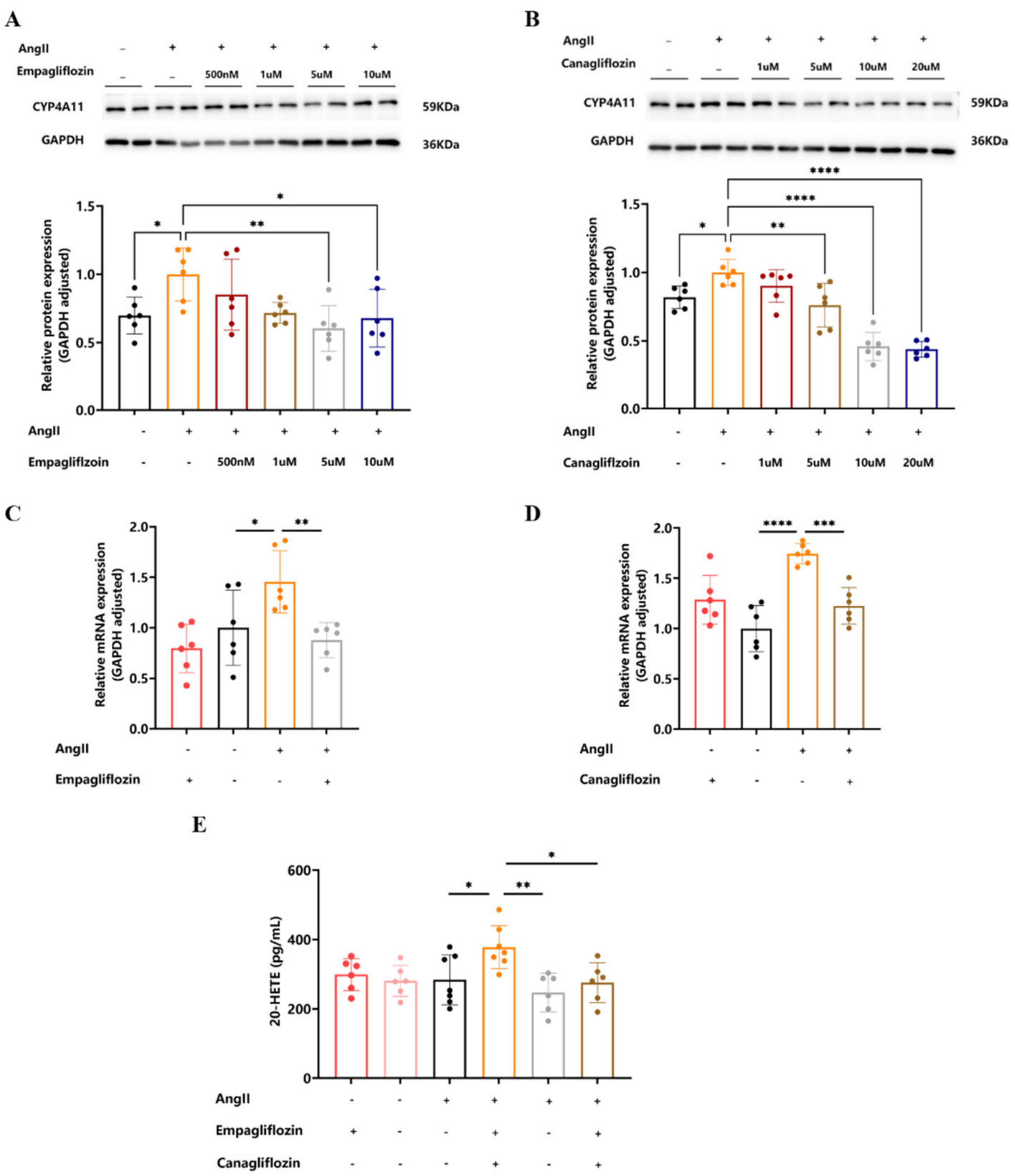

Figure S7. Knockdown efficiency of si-CYP4A.

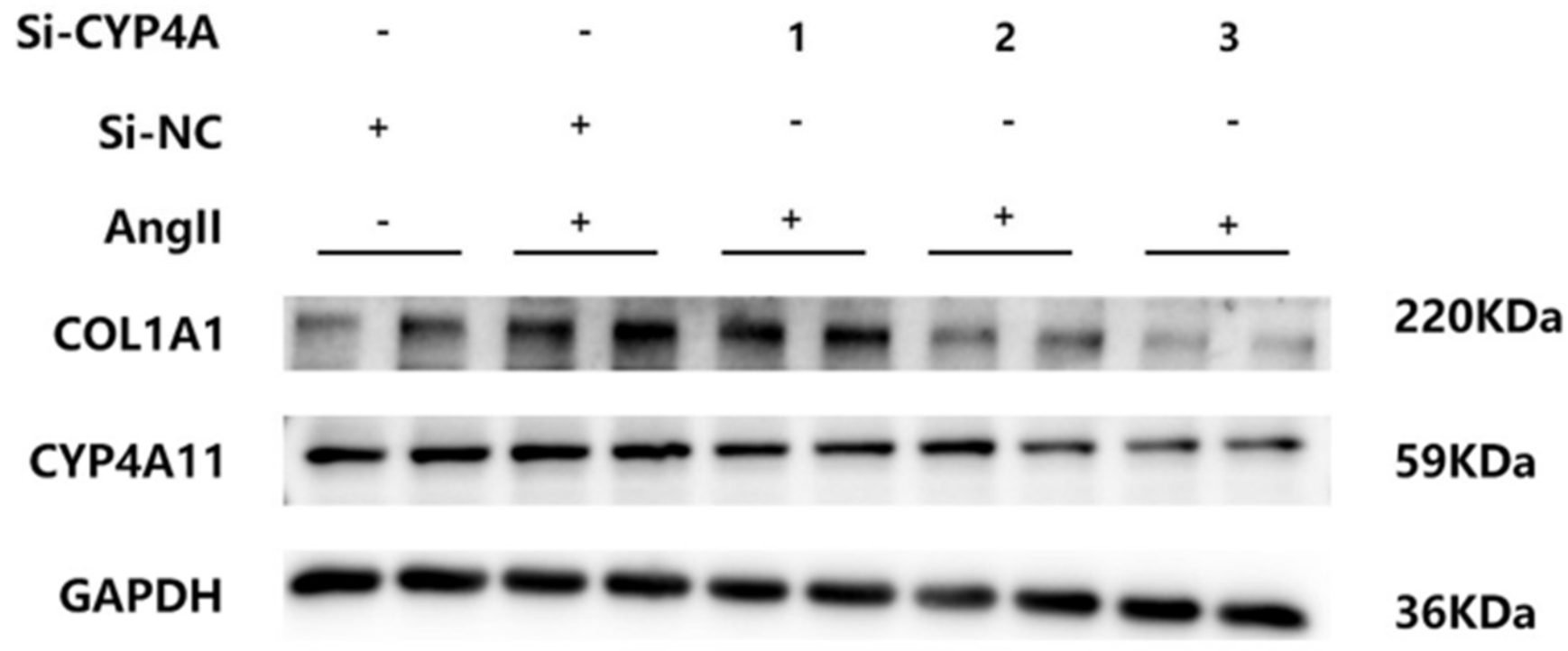

Figure S8. Molecular docking of CYP4A with various SGLT2 inhibitors.

A. The binding affinity of CYP4A with different gliflozins. **B-D**, the 3 dimensional and 2-dimensional molecular docking sites between CYP4A and dapagliflozin.

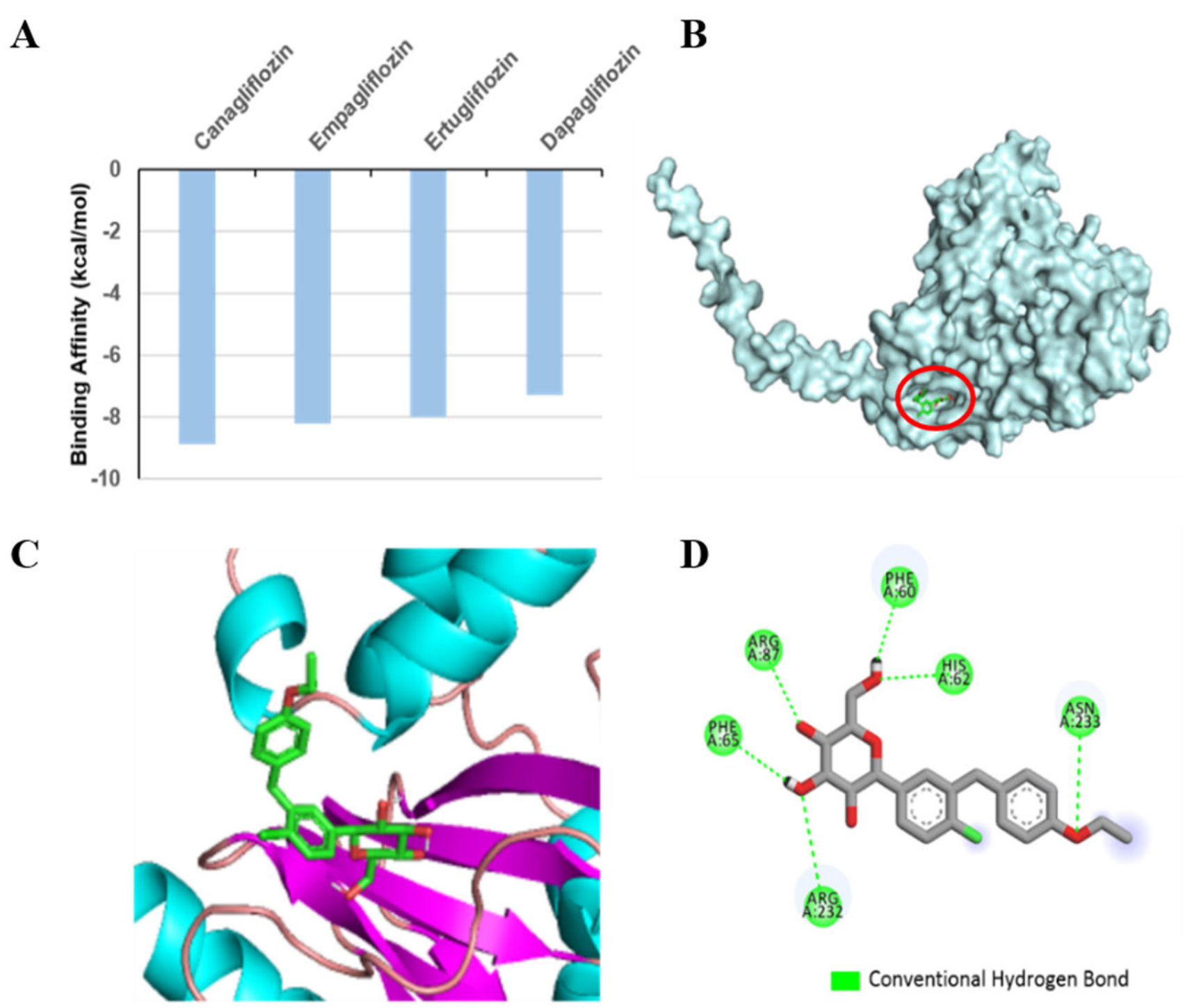

1. Rong R, Hu G, Wang W, Muroya Y, Miura T, Ogawa Y, Kohzuki M, Ito O. Angiotensin II upregulates CYP4A isoform expression in the rat kidney through angiotensin II type 1 receptor. *Prostaglandins & Other Lipid Mediators*. 2018;139:80-86. doi: 10.1016/j.prostaglandins.2018.09.003

Full unedited gel

Full unedited gel for Figure 3G

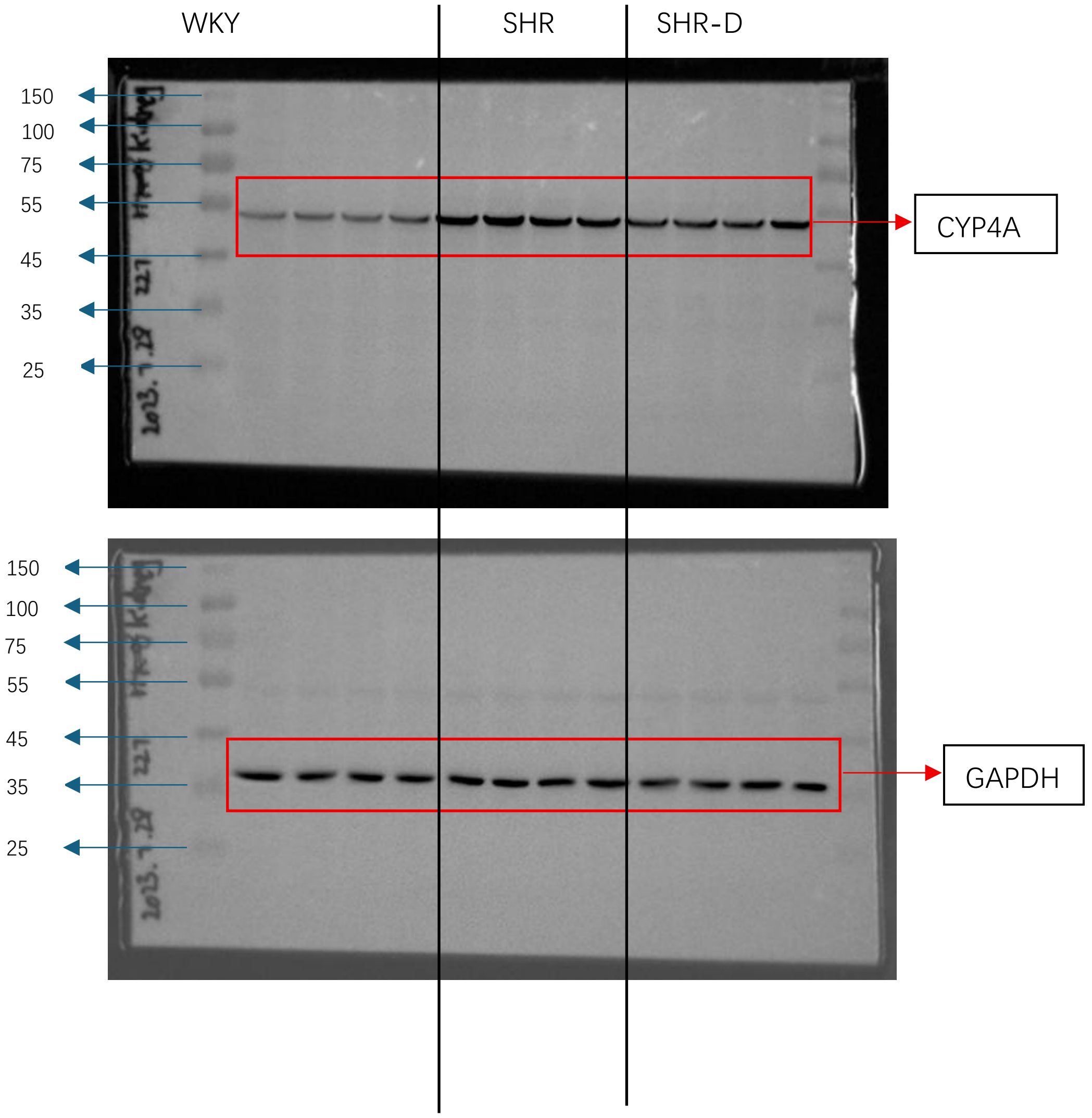

Full unedited gel for Figure 4C

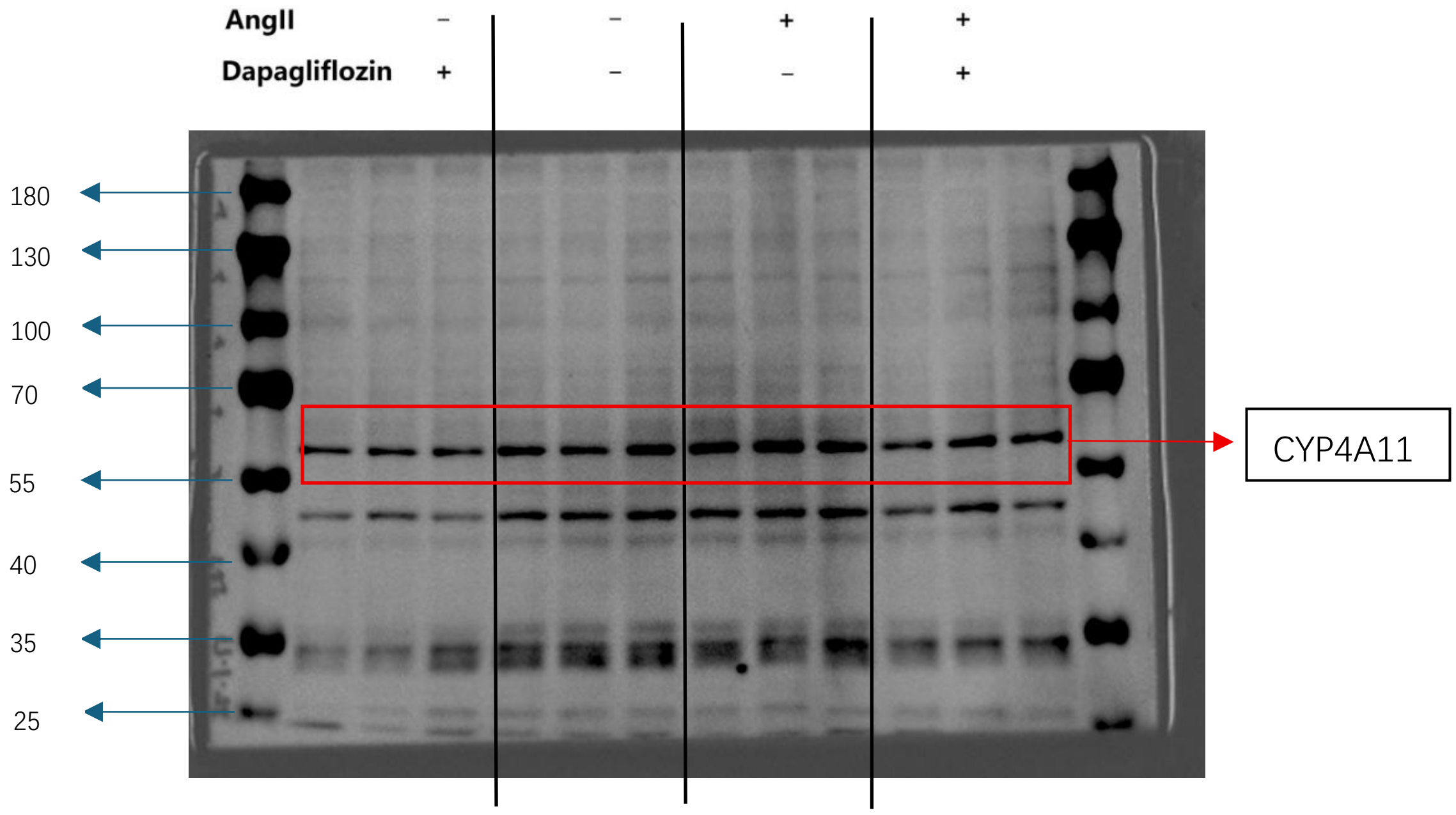

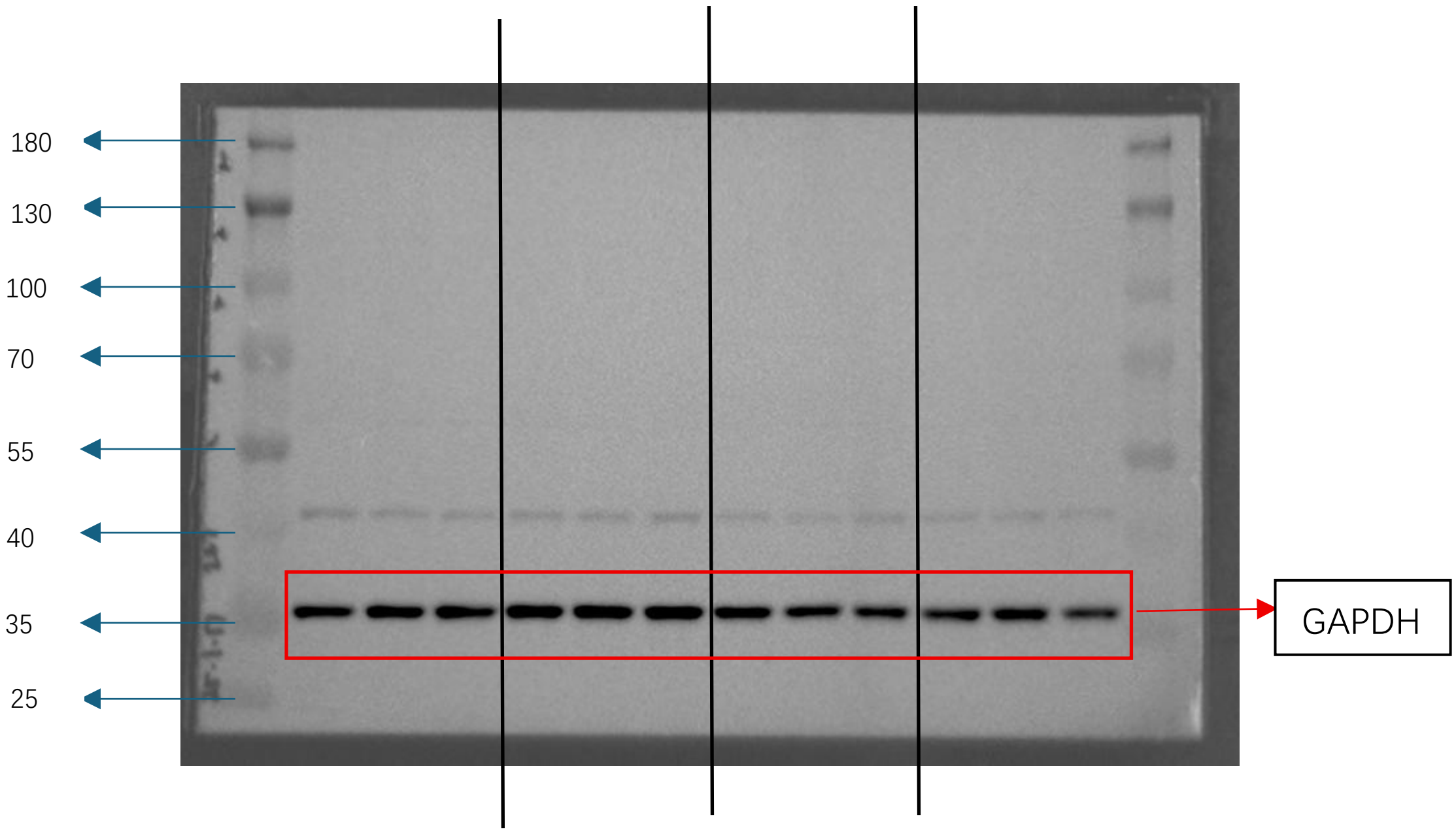

Full unedited gel for Figure S3F

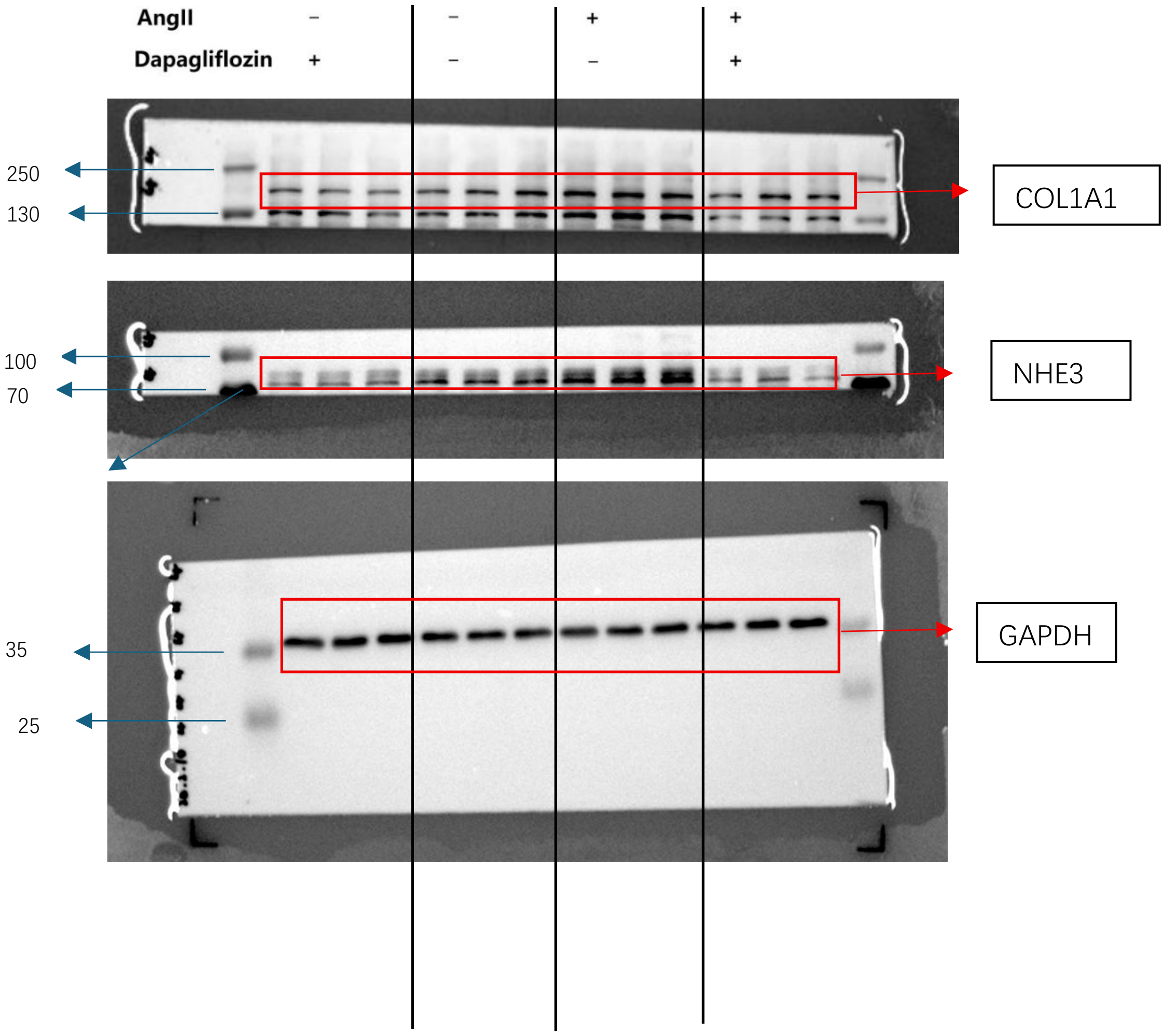

Full unedited gel for Figure S5

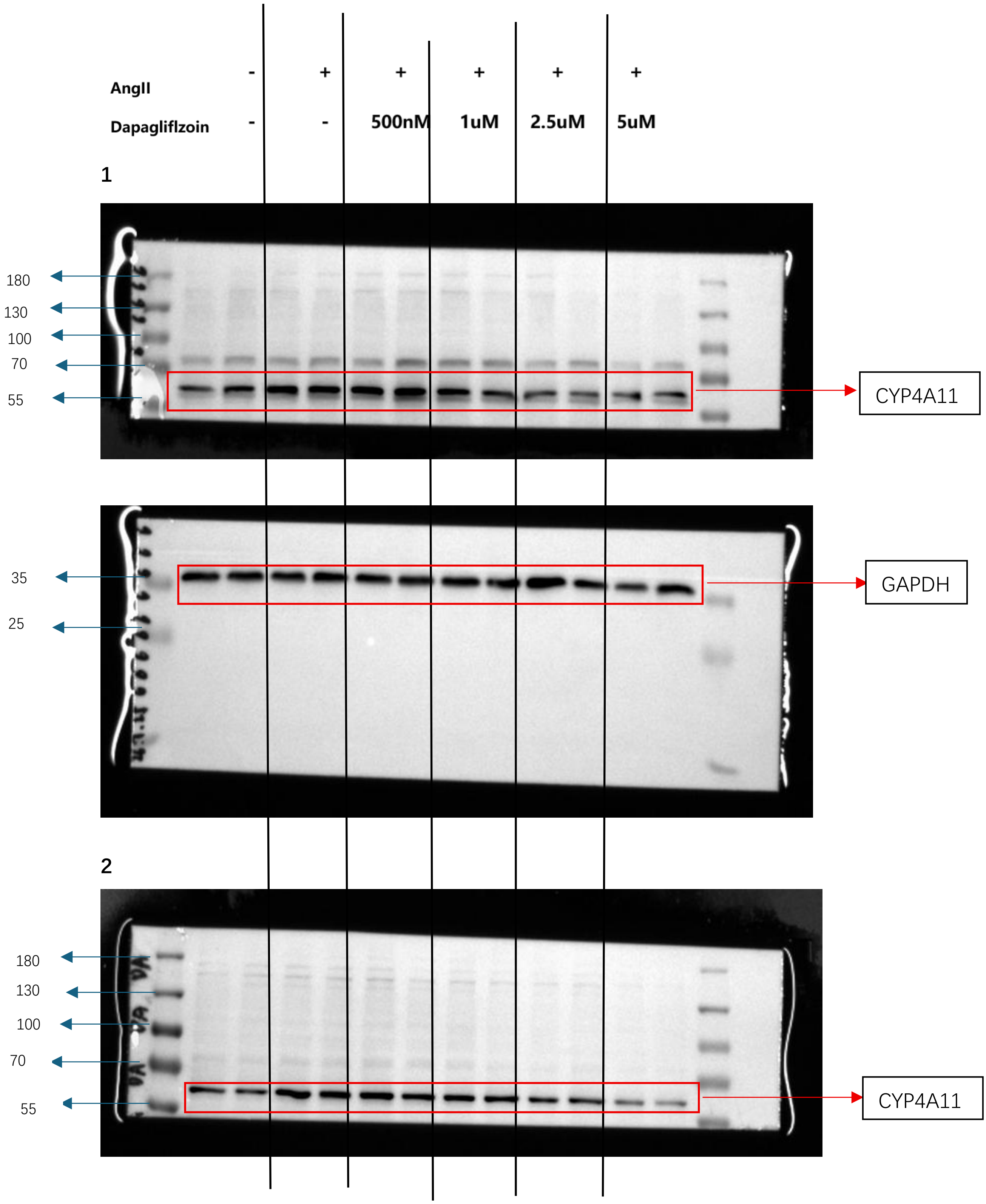

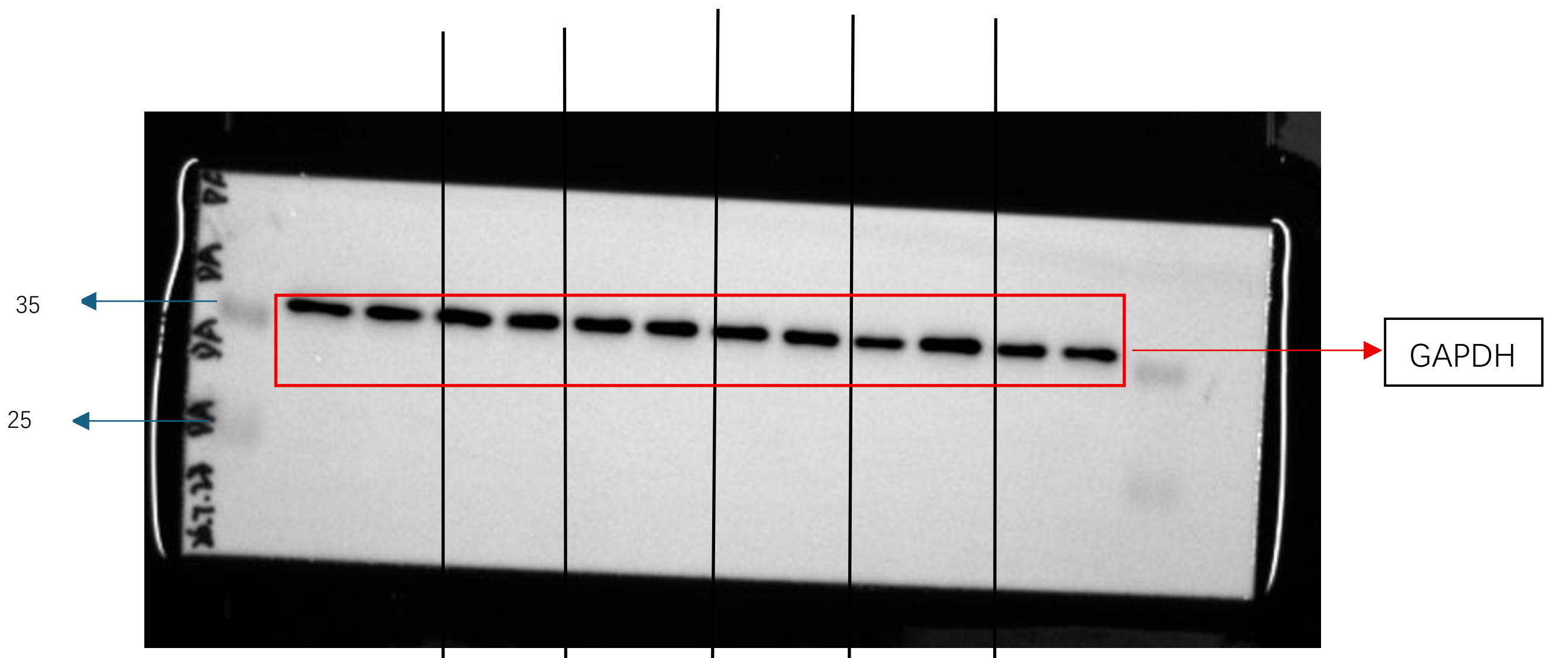

3

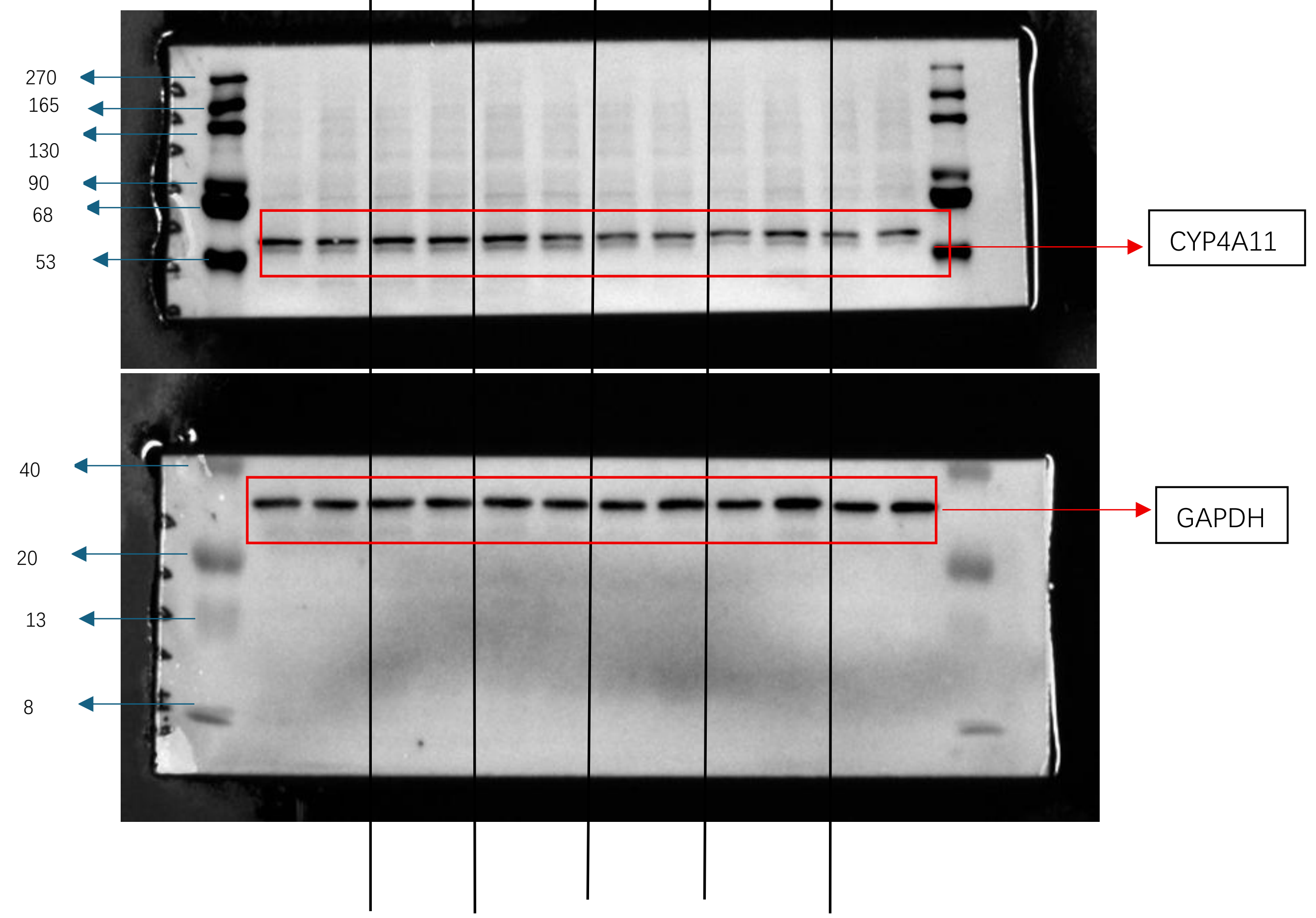

Full unedited gel for Figure S6A

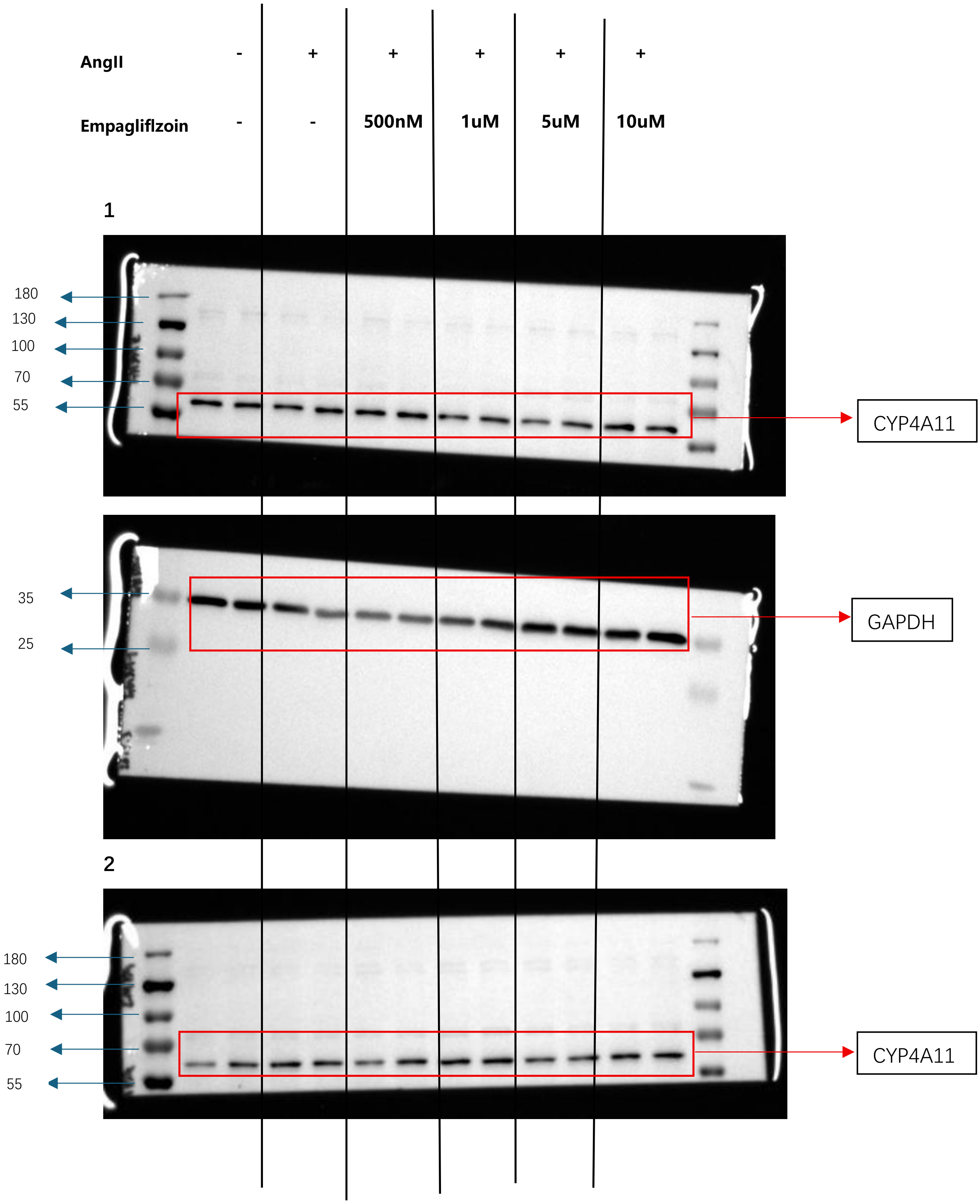

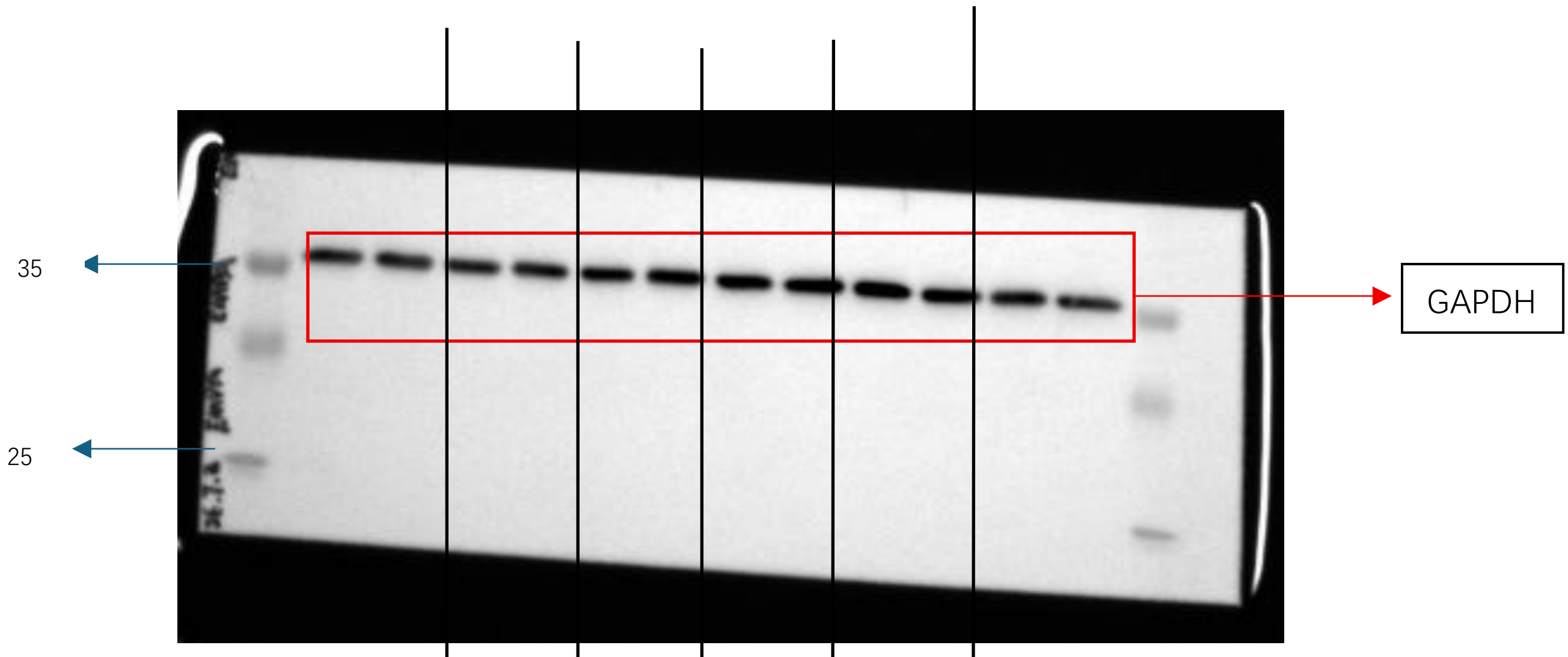

3

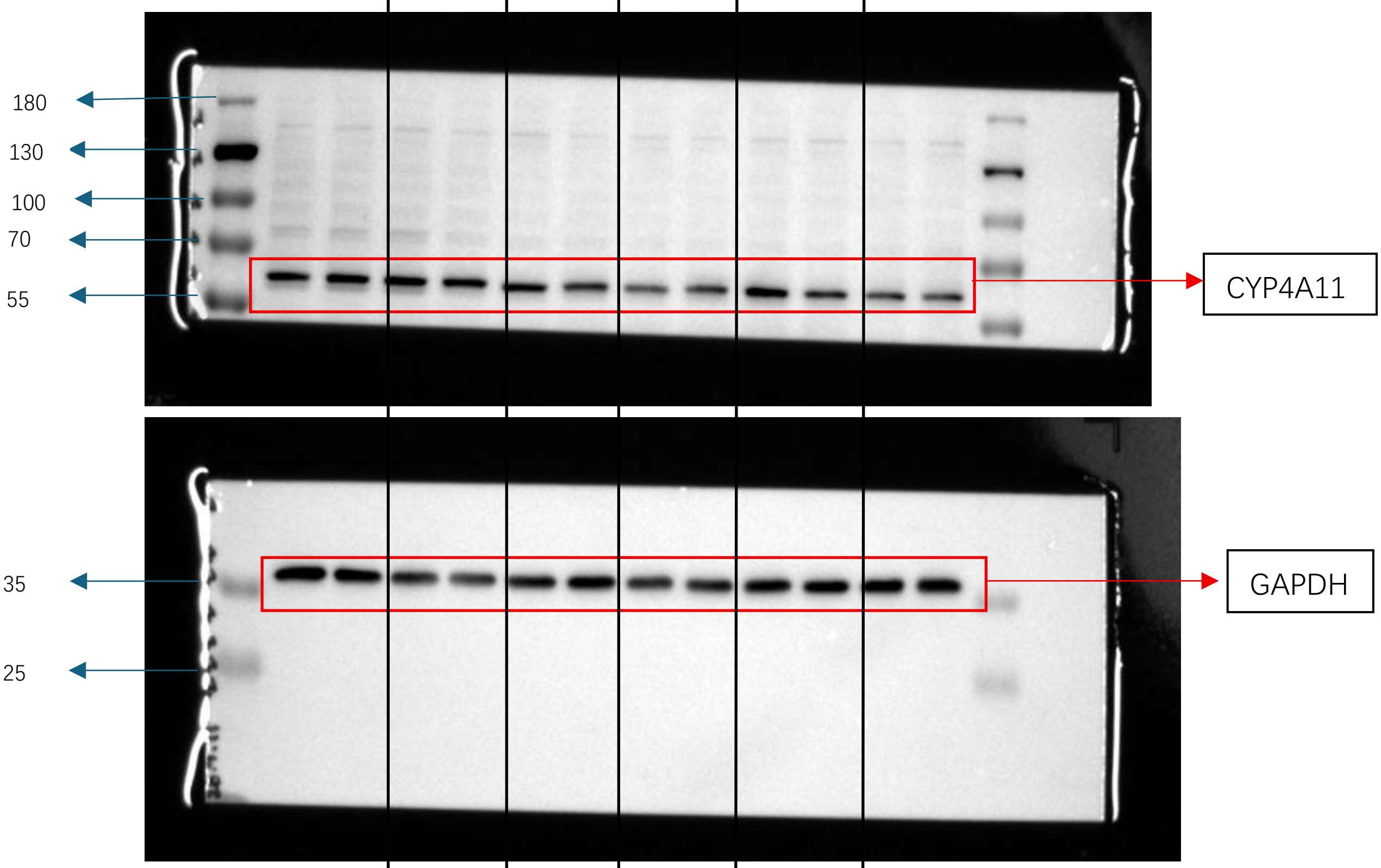

Full unedited gel for Figure S6B

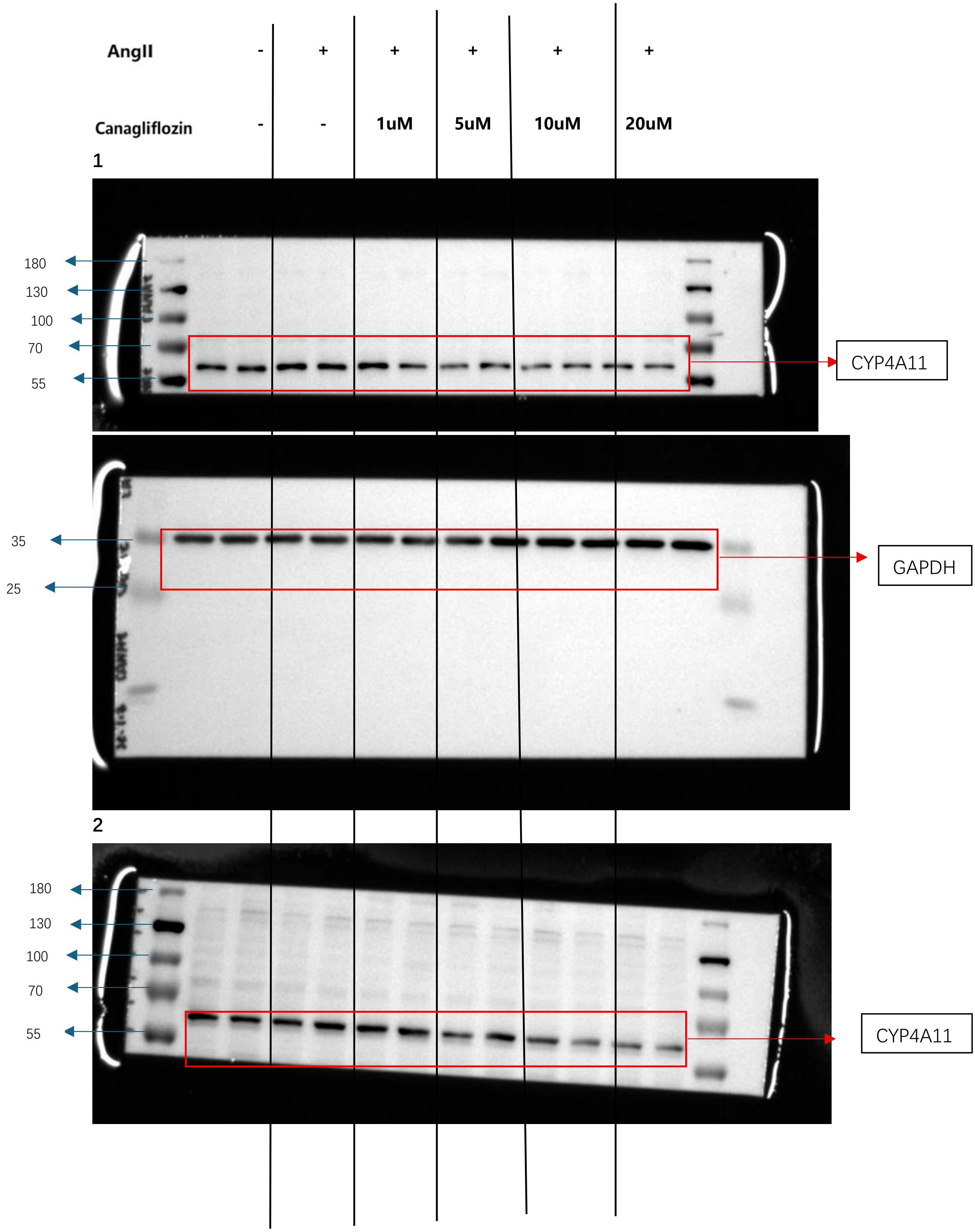

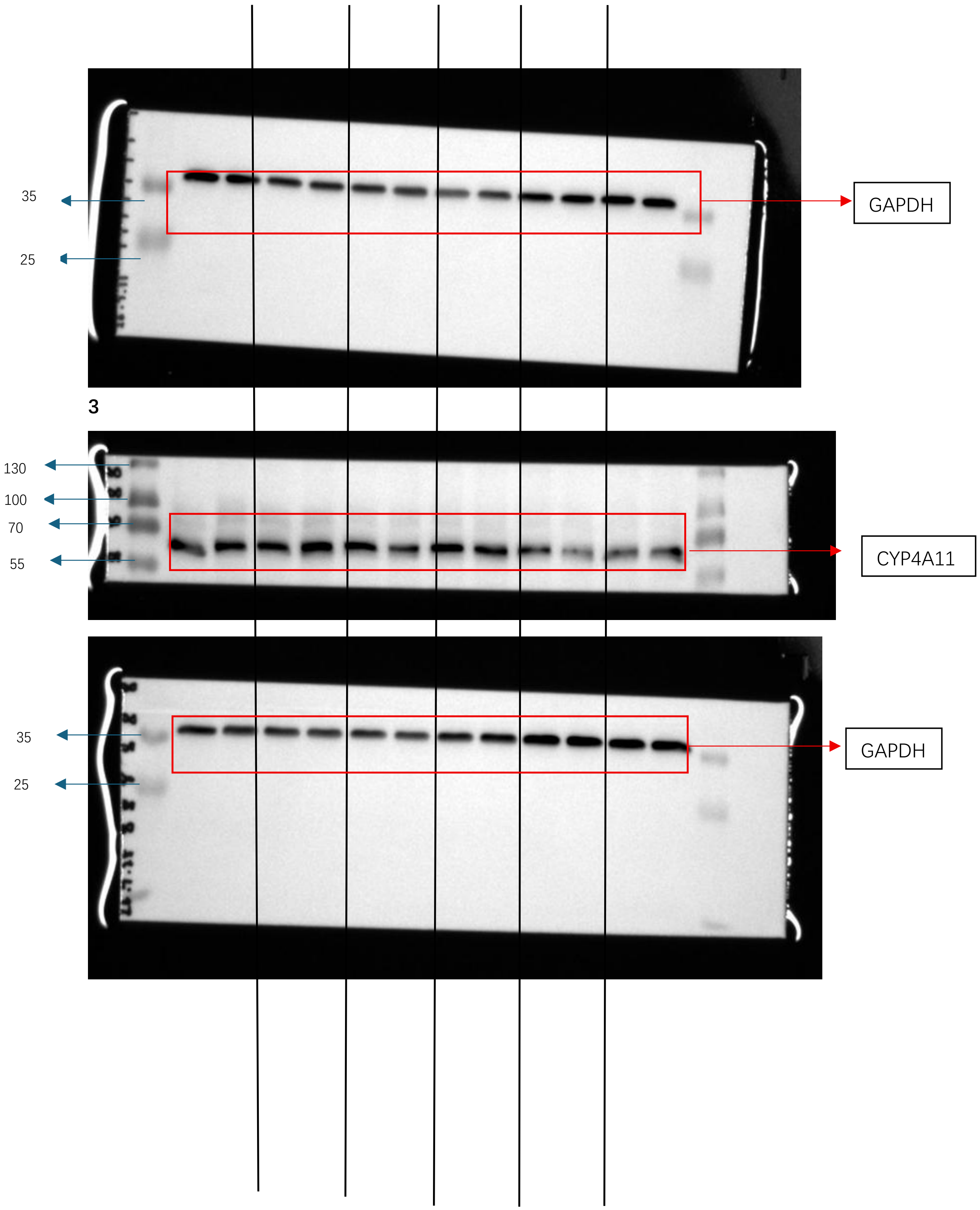
35
25
GAPDH
3
130
100
70
55
CYP4A11
35
25
GAPDH

Full unedited gel for Figure S7

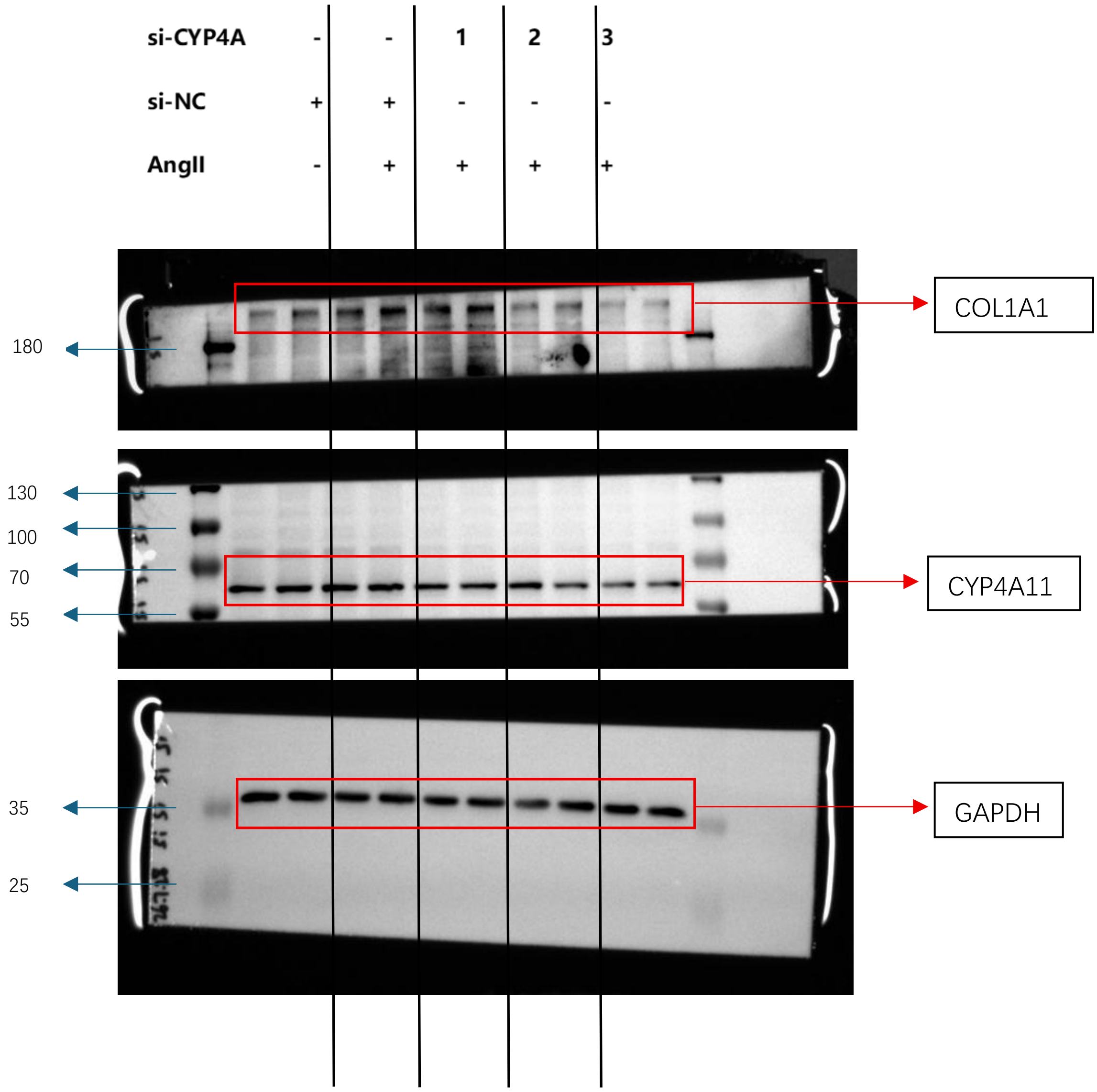

Supplement: Supplementary file 1 [file BSR-2026-0150_supp.pdf]
